# Morphology and mechanism of highly selective Cu(II) oxide nanosheet catalysts for carbon dioxide electroreduction

**DOI:** 10.1038/s41467-021-20961-7

**Published:** 2021-02-04

**Authors:** Xingli Wang, Katharina Klingan, Malte Klingenhof, Tim Möller, Jorge Ferreira de Araújo, Isaac Martens, Alexander Bagger, Shan Jiang, Jan Rossmeisl, Holger Dau, Peter Strasser

**Affiliations:** 1 Department of Chemistry, Chemical Engineering Division, Technical University Berlin, Straße des 17. June 124, 10623 Berlin, Germany; 2 Department of Physics, Free University of Berlin, Arnimallee 14, 14195 Berlin, Germany; 3 European Synchrotron Radiation Facility (ESRF), 38000 Grenoble, France; 4 Department of Chemistry, University of Copenhagen, Copenhagen, 2100 Denmark

**Keywords:** Catalytic mechanisms, Electrocatalysis, Carbon capture and storage, Energy storage

## Abstract

Cu oxides catalyze the electrochemical carbon dioxide reduction reaction (CO2RR) to hydrocarbons and oxygenates with favorable selectivity. Among them, the shape-controlled Cu oxide cubes have been most widely studied. In contrast, we report on novel 2-dimensional (2D) Cu(II) oxide nanosheet (CuO NS) catalysts with high C_2+_ products, selectivities (> 400 mA cm^−2^) in gas diffusion electrodes (GDE) at industrially relevant currents and neutral pH. Under applied bias, the (001)-orientated CuO NS slowly evolve into highly branched, metallic Cu^0^ dendrites that appear as a general dominant morphology under electrolyte flow conditions, as attested by operando X-ray absorption spectroscopy and in situ electrochemical transmission electron microscopy (TEM). Millisecond-resolved differential electrochemical mass spectrometry (DEMS) track a previously unavailable set of product onset potentials. While the close mechanistic relation between CO and C_2_H_4_ was thereby confirmed, the DEMS data help uncover an unexpected mechanistic link between CH_4_ and ethanol. We demonstrate evidence that adsorbed methyl species, *CH_3_, serve as common intermediates of both CH_3_H and CH_3_CH_2_OH and possibly of other CH_3_-R products via a previously overlooked pathway at (110) steps adjacent to (100) terraces at larger overpotentials. Our mechanistic conclusions challenge and refine our current mechanistic understanding of the CO_2_ electrolysis on Cu catalysts.

## Abstract (web summary)

Copper oxides (CuO) can selectively catalyze the electrochemical reduction of CO_2_ to hydrocarbons and oxygenates. Here, the authors study the activity and morphological evolution of 2D CuO nanosheets under applied electrode potentials to conclude the primacy of dendritic shapes and involvement of a new coupling pathway.

## Introduction

Valorizing atmospheric CO_2_ into C_2+_ products by electrochemical method holds the promise to store renewable surplus electricity while closing the global carbon cycle^1–3^. Efficient CO_2_ electrolysis requires advanced electrocatalyst designs^4–10^, an understanding of competing reaction pathways^11,12^, and a reliable device performance at industrial conditions^13–16^. Among a wide variety of catalysts tested for electrochemical CO_2_ reduction reaction (CO2RR), copper-based materials, in particular Cu oxides, CuO_x_, have been enjoying attention thanks to their wide chemical selectivity for a variety of multi-carbon products, such as C_2+_ hydrocarbons and oxygenates. It has been reported that over 16 kinds^17^ of products can be formed on copper catalysts through multiple proton-coupled electron transfer processes.

The cathodic electrode potentials during the catalytic CO2RR invariably drive the chemical transformation of operating CuO_x_ catalysts into metallic Cu phases. This prompted their designation as oxide-derived copper (OD-Cu) catalysts. As a consequence of the chemical reduction metallic Cu^0^, complex time trajectories of catalyst morphology, chemical state, and catalytic selectivity ensues. A full molecular correlation and understanding of these concomitant trajectories has remained elusive. It has been suggested that the increased local pH value^18–20^ of OD-Cu, in direct proportion to the surface roughness, plays an important role for favorable ethylene selectivity compared to methane^21^. Other works highlighted the distinct chemisorption of CO intermediates^22–24^. Yet other reports related the catalytic performance to grain boundaries^25^, to undercoordinated sites^26^, to the presence of subsurface oxygen^27,28^ or to residual near-surface Cu^+^^29^. Also, in most studies, the end point of the morphological evolution of designer OD-Cu catalysts has remained in the dark.

Shape-selected cubic Cu_2_O catalysts—largely obtained through potential cycling in presence of surface-active electrolyte additives—have been receiving particular attention as active and selective CO2RR electrocatalysts^30,31^. Thanks to the structure sensitivity of the C-C bond formation on square atomic Cu^0^ surface lattices and the cubic nature of the Cu_2_O crystal system, the resulting preferential Cu(100) facets offer kinetic faradaic efficiency and onset potential benefits for the formation of C_2+_ products, in particular C_2_H_4_^32^.

To follow the evolution in chemical state and morphology of electrocatalysts under stationary electrode potential conditions, operando X-ray-based^33^ as well as in situ vibrational spectroscopies^31,34–36^, and in situ electrochemical transmission electron microscopy (TEM)^37–39^ were used to record changes in catalyst chemical state^40,41^, local structure^13^, or intermediates^42^. However, to date, real-time tracking individual CO2RR product yields and product onset potentials on OD-Cu catalysts at fast time scales under non-stationary, transient conditions has remained very challenging. This operational mode, however, is very relevant for practical CO2RR electrolyzers powered by intermittent input electricity from renewable sources.

Even though Cu-based CO2RR electrocatalysts have shown excellent C_2_H_4_ efficiencies in highly alkaline pH 13–15 conditions^13,43^, practical CO2RR electrocatalysis must operate at neutral pH ~ 7 conditions and perform at industrially relevant current densities > 200 mA cm^−2^^13,14^. To achieve this, the catalysts must be cast in gas diffusion electrodes (GDEs) that are deployed in single zero- and non-zero gap flow electrolyzer cells. While a few recent studies have reported the use of free-standing cubic Cu_2_O nanocubes^44–46^ inside GDE designs, the majority of OD-Cu based GDE studies relied on top-down approaches involving modified bulk Cu^43^. Compared to bulk Cu-based catalysts, nanostructured free-standing catalysts are more desirable as they exhibit higher surface-to-volume ratio and are more amenable to assembly in large-scale GDEs.

In this contribution, we report on a new family of free-standing 2-dimensional Cu(II) oxide electrocatalysts for the CO2RR under neutral pH conditions. Owing to their 2D nature, Cu(II)O nanosheets (referred to as “2D CuO NS”) feature a highly preferred (001) facet orientation. We report a facile one-step synthesis of 2D CuO NS and then track their unique catalytic reactivity and concomitant morphological evolution under applied electrode potentials using in situ electrochemical liquid TEM and operando X-ray absorption spectroscopy (XAS). Next, using a new differential electrochemical mass spectrometry (DEMS) technique with millisecond resolution, we discuss previously unreported time evolutions of kinetic onset potentials of a range of different products over the course of hours. Conclusions from our DEMS data challenge and refine our current mechanistic understanding of the mechanistic link between CH_4_ and ethanol (EtOH). Finally, we document the favorable CO2RR performance of 2D CuO NS inside GDEs of commercial electrolyzers under industrially relevant neutral pH conditions. Owing to their emerging stable dendritic structure, their high catalytic reactivity, and the resulting performance stability, oriented 2D CuO NS precursor catalysts constitute an interesting alternative to conventional cubic Cu_2_O catalysts for the electrochemical conversion of CO_2_ into C_2_H_4_.

## Results

### Synthesis and characterization of 2-dimensional CuO nanosheets

**Fig. 1:**
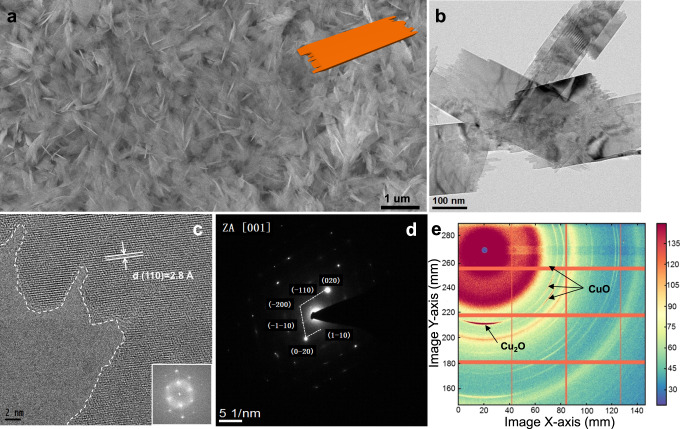
Morphological, structural characterizations of sheet-like CuO catalysts synthesized in this study by ex situ techniques. **a** Large-scale scanning electron microscopy (SEM) image and 3D structure of CuO NS (insert, orange). **b** Transmission electron microscopy (TEM) image and **c** high resolution TEM (HR-TEM) images with measured lattice distance and the corresponding fast Fourier transformation. **d** Selected area electron diffraction (SAED) pattern along zone axis [001]. **e** 2D Synchrotron grazing incidence wide-angle X-ray scattering (GI-WAXS) image demonstrating preferred orientation of as-prepared CuO NS on glassy carbon. Additional azimuthally integrated line profiles are shown in Supplementary Fig. S5b.

Free-standing CuO nanosheet catalysts (CuO NS) were prepared by solvothermal formation and processing of Cu(OH)_2_ intermediates in alkaline condition (Supplementary Fig. S2). The wrinkled thin Cu(OH)_2_ layers showed high aspect ratios prior to decomposing into CuO and H_2_O. Figure 1a,b presents scanning electron microscopy (SEM) image and transmission electron microscopy (TEM) image of as-prepared CuO NS. The 2D character of the material was retained during the thermal decomposition of Cu(OH)_2_, while larger sheets split into small ones. The final rectangular CuO NS exhibited serrated edges on the short edges. Supplementary Fig. S3a indicates a stacked structure of individual free-standing CuO NS of 3−4 nm thickness. High-resolution transmission electron microscopy (HR-TEM) (Fig. 1c) of CuO NS revealed well-defined lattice fringes with an interplanar spacing of 2.8 Å, corresponding to {110} planes of monoclinic CuO. Selected area electron diffraction (SAED) images (Fig. 1d) showed rhombic diffraction spots along the [001] zone axis. This can be seen by indexed (020), (-110), (-200), (-1-10), (0-20) and (1-1;0) planes, indicating that the CuO NS had {001} exposed surfaces. The relative intensity of the diffraction spots is presented in Supplementary Fig. S3b. The crystal structure is provided in Supplementary Fig. S4 with indexed (001), (110) and (11-1) planes.

A 2D grazing-incidence wide-angle X-ray scattering (GI-WAXS) pattern of CuO NS on glassy carbon electrode is shown in Fig. 1e. The pattern is commensurate with a crystalline CuO phase. Minor contributions of a residual Cu_2_O phase are seen at low angles, yet all higher order reflections were absent evidencing their trace character. Importantly, the (002) and (11-1) reflections of CuO exhibited a stronger intensity in the meridional direction, evidencing that the CuO NS are stacked along the <00 l> direction, supporting the HR-TEM results. A partial contour plot and integrated CuO NS line profiles are shown in Supplementary Fig. S5, indicating the coexistence of Cu_2_O traces and major CuO phase.

### CO2RR reactivity, stability, and ex situ morphology in H-cells

**Fig. 2:**
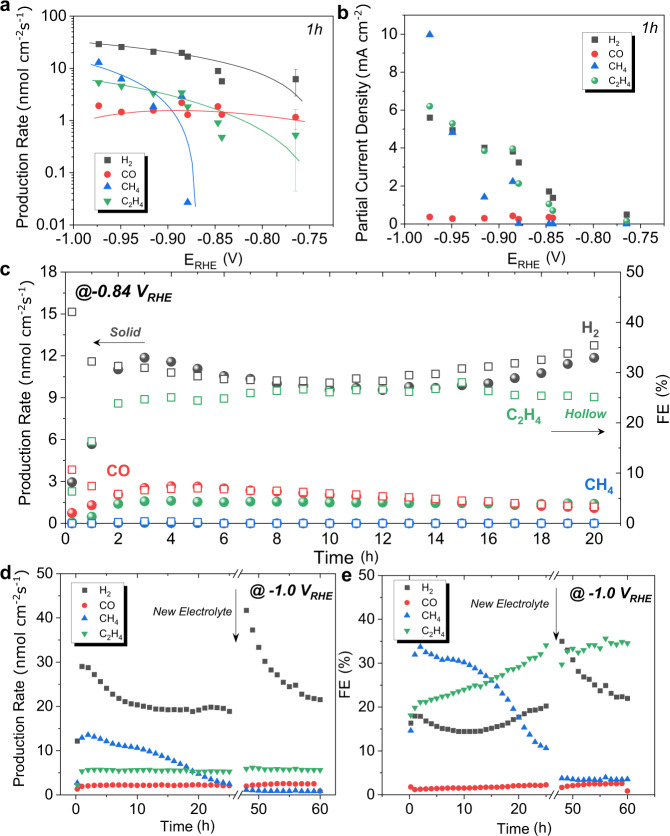
Electrocatalytic CO2RR tests using CuO NS in H-cells. **a** Absolute product formation rates of major gaseous products as a function of applied electrode potentials during CO2RR in CO_2_-saturated 0.1 M KHCO_3_ at 60 min. **b** Partial current densities as a function of applied electrode potentials during CO2RR in CO_2_-saturated 0.1 M KHCO_3_ at 60 min. **c** Chronoamperometric performance stability of the CO_2_ reduction reaction on CuO NS in CO_2_-saturated 0.1 M KHCO_3_ at −0.84 V_RHE_. **d** and **e** Long-term stability test over 60 h for **d** absolute product formation rates of major gaseous products and **e** Faradaic efficiencies on CuO NS in CO_2_-saturated 0.1 M KHCO_3_ at −1.0 V_RHE_. The error bars are given as standard error of mean. Catalyst loading: 100 μg cm^−2^.

The catalytic CO2RR activity of the CuO NS in pH neutral conditions and without any further pretreatment was recorded for up to 60 h. Product yields as function of iR-corrected electrode potential (E_RHE_) after 1 h electrolysis are plotted in Fig. 2a. C_2_H_4_ started to evolve at significantly more anodic electrode potentials compared to CH_4_, which offered a roughly 300 mV-wide potential window of exclusive C_2_H_4_ production (their detailed time-resolved onset potentials see DEMS section below). CH_4_ formation showed a rapid growth beyond −0.9 V_RHE_ and exceeded C_2_H_4_ production at −0.97 V_RHE_. The observed partial current densities under the chosen conditions (Fig. 2b) are testament to previously unachieved hydrocarbons selectivities under pH neutral conditions. The partial current density of C_2_H_4_ reached 6.2 mA cm^−2^ at −0.97 V_RHE_, while CO remained at very low levels during the entire given overpotential range. The Faradaic efficiencies (FEs) within the potential window of exclusive C_2_H_4_ production and beyond are compared in Supplementary Fig. S6. The suppression of the HER and concomitant increase in C_2_H_4_ are noteworthy for H-cell studies.

Longer-term 20 h and 60 h electrolysis was carried out to track the CuO NS selectivity and stability at selected potentials (Fig. 2c−e). CuO NS catalysts maintained a stable absolute C_2_H_4_ production rate during the 60 h electrolysis, even past replacements of the electrolyte (Fig. 2d, e). By contrast, the CH_4_ production decreased and dropped to very low values after 25 h, making CuO NS voltage efficient (low overpotentials), C_2_H_4_-selective and very performance-stable electrocatalysts.

The initial catalyst activation period over the first few hundred minutes merits a closer time resolution of the reactivity coupled to a correlation to the sheet morphology (Supplementary Figs. S7−S10). The time trajectories of CO, CH_4_, and C_2_H_4_ production (Supplementary Fig. S7) exhibited similar patterns over the first 2−3 h of CuO NS catalyst activation. The hydrocarbon production rate peaked earlier with increasing applied overpotential. Consistent with data from Fig. 2a, exclusive and sustained catalytic C_2_H_4_ production was detected at −0.84 V_RHE_ at 1.6 nmol cm^−2^ s^−1^, exceeding previous reports^47^. Near −1.0 V_RHE_, hydrocarbon production peaked already after 2 h, followed by a steady drop in CH_4_ generation (cf. Fig. 2d).

Morphological changes of CuO NS (Supplementary Fig. S8) after the first hour electrolysis near −1.0 V_RHE_ involved rounding of the nanosheet with agglomerations. After prolonged electrolysis at −0.76 V_RHE_ and −0.84 V_RHE_ (Supplementary Figs. S9, S10), the rounding led to a sheet fragmentation of the initial CuO NS appeared to be more obvious. Finally, after a 60-h electrolysis at −1.0 V_RHE_, the sheet fragments re-assembled into larger agglomerates with rough surfaces (Supplementary Fig. S11). In a comparative study of the evolution of the catalyst morphology as a function of electrode potential under identical conditions, the CuO NS catalyst was drop cast on a rough carbon fiber paper rather than on smooth glassy carbon. Thanks to better dispersed NS, the catalytic current density increased (Supplementary Fig. S12). Now, ex situ SEM studies after 1 h reaction (Supplementary Fig. S13) evidenced that rough supports slowed down the morphological transformations of the CuO NS. Unlike shown in Supplementary Fig. S8, part of the sheet morphology appears still intact (Supplementary Fig. S13c, Area 3), with individual sheets on the fiber cracked (Supplementary Fig. S13b, Area 1) and fractured into small clusters (Supplementary Fig. S13b, Area 2).

### Real-time morphology using in situ electrochemical TEM—from sheets to dendrites

The ex situ microscopic studies revealed rapid (< 1 h) fragmentation of CuO NS during CO2RR associated with rapid kinetic activation and monotonically rising product selectivity (Supplementary Figs. S7, S8 and S13). Of particular interest was the applied bias of −0.84 V_RHE_, where hydrocarbon generation was limited exclusively to C_2_H_4_. To learn more about the NS catalyst morphology that enabled exclusive C_2_H_4_-selectivity, we followed the transformations of the CuO NS at that same bias using in situ electrochemical liquid TEM experiments. These studies were carried out in a Protochips Poseidon holder that hosted a flow-through electrochemical chip cell (“in situ TEM E-chip cell”, Supplementary Fig. S14b) equipped with parallel beam-transparent silicon nitride windows. The top view of the in situ TEM E-chip cell is illustrated in Supplementary Fig. S14a. To exclude beam damage and liquid layer effects, all in situ TEM E-chip cell experiments were cross-checked using identical location (IL) TEM E-chip cells, in which the E-chip cell was operated outside the microscope under otherwise identical conditions (Supplementary Fig. S14c), yet imaged in the dry state. The in situ studies cover the first few minutes of the catalyst activation where the most dramatic rise in catalytic activity occurred (Supplementary Fig. S7).

**Fig. 3:**
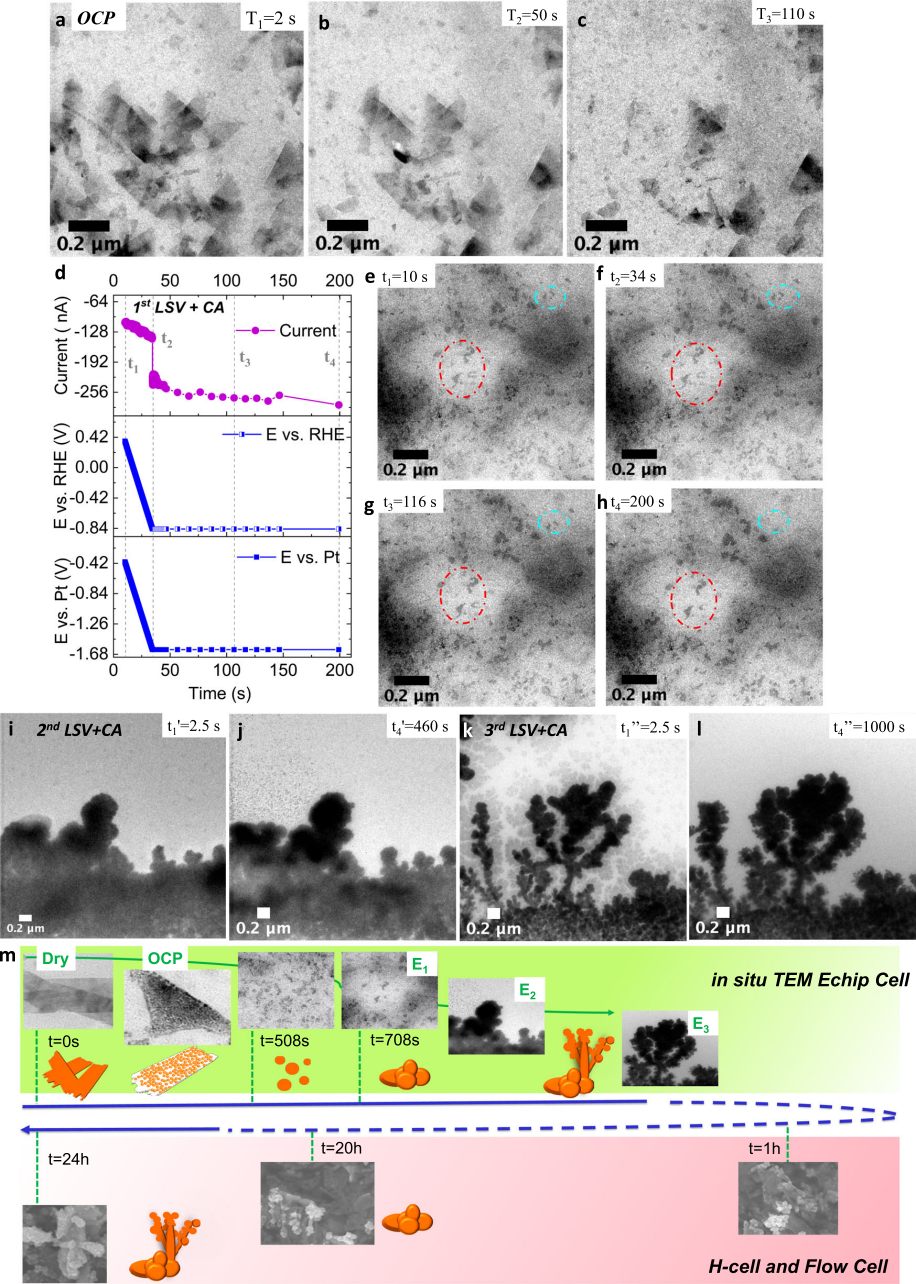
Real-time imaging of CuO NS catalysts under electrochemical reaction. In situ TEM imaging of catalyst structure during OCP in 30 µL/h H_2_O flow at **a** 2 s, **b** 50 s and **c** 110 s. The corresponding OCP profile is presented in Supplementary Fig. S15. The whole movie is shown as Supplementary Movie 1. **d** Current/potential profiles over time with marked time points, t_1_ - t_4_ (vertical lines), corresponding to the images in **e**–**h**. Linear sweep voltammetry (LSV) is performed after 10-second OCP measurement in a pH = 6.9 buffer solution flow (the OCP profile is given in Supplementary Fig. S17a) with scan rate of 50 mV/s. The following Chronoamperometry (CA) are acquired at −0.84 V_RHE_ (the first potential). The whole movie is shown as Supplementary Movie 4. The corresponding images at **i** $t_1^\prime$ = 2.5 s and **j** $t_4^\prime$ = 460 s with the second LSV + CA (the 10-second OCP profile before LSV is given in Supplementary Fig. S17b) at −1.23 V_RHE_. Current/potential profiles over time with marked time points, $t_1^\prime$ - $t_4^\prime$ (vertical lines) and the images snapshots are shown in Supplementary Fig. S18. The whole movie is shown as Supplementary Movie 5. The corresponding images at **k** $t_1^\prime\prime$ = 2.5 s and **l** $t_4^\prime\prime$ =1000 s with the third LSV + CA (the 10-second OCP profile before LSV is given in Supplementary Fig. S17c) at −1.73 V_RHE_. Current/potential profiles over time with marked time points, ${t_1^\prime}\prime$ - $t_4^\prime\prime$ (vertical lines) and the images snapshots are shown in Supplementary Fig. S19. The whole movie is shown as Supplementary Movie 6. **m** Schematic overview (time line) of the experimentally observed evolution of the CuO NS morphology probed by the in situ TEM E-chip flow cell, H-cell, and flow cell electrolyzer.

First, Supplementary Movie 1 tracks the initial morphological evolution of the CuO NS under open circuit potential (OCP) and pH-neutral aqueous conditions. Three selected times (Supplementary Fig. S15) and their TEM snapshots are shown in Fig. 3a–c. CuO NS of several 100 s of nm in size fracture into smaller sheet fragments, however without immediate disintegration. Over the 110 s imaging time, some of the fragmented sheets slowly drifted out of view into the liquid layer.

To check the influence of beam and liquid layer effects, we conducted a prolonged 40 min OCP experiment in the IL TEM E-chip cell. In agreement with the in situ results, the CuO NS with 100 s of nanometer size fractured into fragments of few nanometer size (Supplementary Fig. S16b, c) without disintegration.

Next, we used the in situ TEM E-chip cell to conducted real-time imaging of the CuO NS in the pH = 6.9 buffer solution, still maintaining OCP. Supplementary Movies 2 and 3 captured the structural transformations over the next ~ 400 s: Supplementary Movie 2 documents the continued fragmentation of the CuO NS in real time at OCP over 200 s. Clouds of dark spherical Cu fragments are distributed non-uniformly across the field of view, while floating and drifting in and out of focus. Over the next 198 s, Supplementary Movie 3 captures the dynamics of the fragmented CuO NS catalyst. We believe that these sheet fragments served as building blocks for later re-agglomeration. Next, we investigated the effect of applied electrode bias of −0.84 V_RHE_ on the subsequent morphological evolution of the fragmented Cu catalyst. After 10 s OCP (Supplementary Fig. S17a), a linear sweep voltammetry (LSV) step lowered the electrode potential from +0.42 V_RHE_ to −0.84 V_RHE_, where chronoamperometric (CA) control kept it for another 170 s. Figure3d shows the experimental current/potential profiles recorded inside the in situ TEM E-chip cell. Supplementary Movie 4, from which 4 TEM snapshots at times t_1_- t_4_ (Fig. 3e–h) were taken, evidenced that Cu fragments now start to agglomerate (red circles in Fig. 3e–h), while others vanish (cyan circles). Finally, after another 8 min (Supplementary Movie 5) and yet another 16 min (Supplementary Movie 6) under negative bias, the gradual agglomeration of Cu sheet fragments into initially spherical, then into branched dendritic structures set in, evidenced at time point 65 s and 127 s of Supplementary Movie 5 and 6, respectively. The corresponding in situ TEM snapshot sequence is shown in Fig. 3i–l. Supplementary Movie 5 demonstrates a spherical deposition and growth of sheet fragments from the liquid layer onto Cu nuclei attached to the electrode in the lower portion of the field of view. Supplemental Movie 6 tracked the deposition of Cu sheet fragments onto pre-formed highly branched Cu dendrites with previously unachieved clarity and details. Again, verification of the in situ TEM results ensued using our IL TEM E-chip cell. Supplementary Fig. S20 contrasts oriented 2D CuO NS morphologies with the final Cu dendrite morphology after 1600 s at −0.8 V_RHE_ validating the real time studies. We conclude that facet-orientated CuO NS rapidly transform into nanoscale reactive sheet fragments that slowly re-assemble into stable large agglomerations and tree-like Cu dendrites at rates depending on applied potential/reaction time. Dendritic structures are known to emerge in diffusion-limited growth regimes^48,49^, typically present under convective flow conditions of electrochemical flow cells. We therefore hypothesize dendritic structures—characterized by their unique tree-like arborization with self-similar branch structure across several length scales—constitute the kinetically preferred morphological end point of our Cu catalysts in flow conditions;^50^ a hypothesis that will be corroborated further below in our electrolyzer flow cell studies below.

### Tracking the chemical state and local coordination of CuO NS using operando XAS

**Fig. 4:**
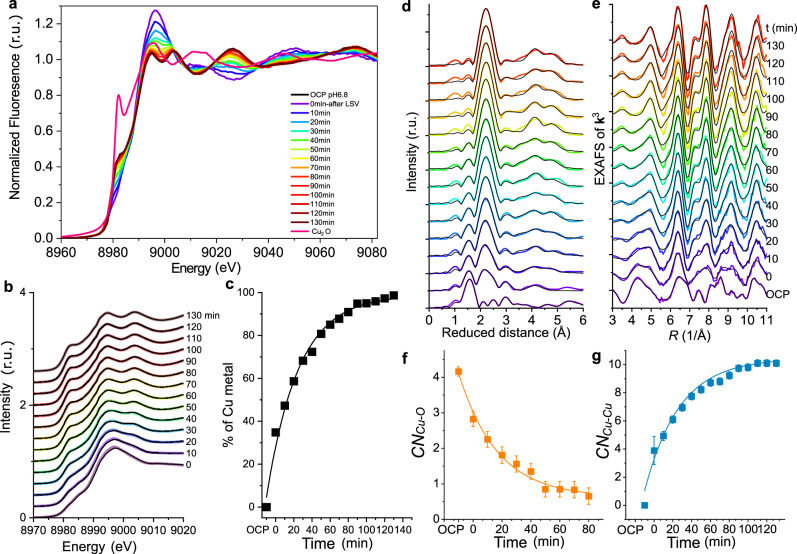
Catalyst evolution during CO2RR characterized by operando XAS. **a** XANES at the Cu K-edge of the CuO NS catalysts at OCP in 0.1 M KHCO_3_ at pH 6.8 during 130 min CO2RR at −0.84 V_RHE_. **b** Linear combinations (with the weighting factors as fit parameters) of Cu foil and the CuO NS catalysts at OCP were fitted to experimental data of the CuO NS catalysts during 130 min CO2RR at −0.84 V_RHE_. Colored lines represent the experimental data and black lines the linear combinations. **c** Amount of Cu metal in the CuO NS catalysts during 130 min CO2RR at −0.84 V_RHE_. Values are obtained by linear combinations (with the weighting factors as fit parameters) of Cu foil and the CuO NS catalysts at OCP, which were fitted to experimental data. **d** FT of **k**^3^-weighted EXAFS at the Cu K-edge of the CuO NS film at OCP and during 130 min CO2RR at −0.84 V_RHE_ in 0.1 M KHCO_3_ at pH 6.8. Colored lines represent the experimental data and black lines the simulations. The distance on the x-axis is reduced by 0.35 Å relative to the real internuclear distance. **e** EXAFS (**k**^3^ weighted) at the Cu K-edge of the CuO NS catalysts at OCP and during 130 min CO_2_RR at −0.84 V_RHE_ in 0.1 M KHCO_3_ at pH 6.8. Colored lines represent the experimental data and black lines the simulations. Coordination numbers of the first Cu-O **f** coordination sphere and the first intermetallic Cu-Cu **g** shell (Details of the fit error and Fourier-filtered error can be found in the Supplementary Information). Catalyst loading: 100 μg cm^−2^.

To examine the changes in the chemical state and local coordination environment of the CuO NS concomitant to the morphological evolution, operando XAS experiments were conducted (Fig. 4).

Visual inspection of X-ray absorption near edge spectra (XANES) of reference materials and CuO NS catalyst under OCP conditions (Supplementary Fig. S22) corroborated earlier findings as to the Cu(II) state of the CuO NS catalyst. Extended X-ray absorption fine structure (EXAFS) simulations proved that the CuO reference and the CuO NS catalyst shared the same atomic structure (Supplementary Fig. S23 and Supplementary Table S1). There were no significant contributions from Cu(OH)_2_ and Cu_2_O in the CuO NS film detected, which is owed to the volume sensitivity of XAS. Hence, the catalyst structure at rest conditions was indeed a highly oriented CuO sheet.

After voltammetric scanning to −0.84 V_RHE_, the complete chemical reduction to metallic Cu^0^ was observed after 130 min during CO2RR. Interestingly, the reduction from cupric oxide, CuO, to Cu^0^ occurred directly, i.e. without detectable formation of Cu_2_O intermediates (Fig. 4a). Linear combination fittings revealed that after the voltammetry, 35% of the bulk volume was already Cu^0^ (Fig. 4b, c). At the end of the chronoamperometry, metallic Cu accounted for 99%, with the remaining 1% within the uncertainty of the data quality. Possible effects of residual traces of subsurface oxygen or higher valent Cu species can neither be excluded nor confirmed^33^, yet appear unlikely in absence of stabilizing agents such as halogenides. The gradual change from CuO to Cu^0^ is also indicated by the intensity decrease of the Cu-O bond (1.94 Å) and the intensity increase of the Cu-Cu (2.53 Å) bond (Fig. 4d, e). Coordination numbers (*CN*) of Cu-O decreased with the reduction process, while CN of Cu-Cu increased, and stabilized at 10 after 120 min (Supplementary Fig. S25). There is neither variation in the Cu-O bond length (first coordination sphere), nor in that of Cu-Cu. The obtained *CN* of Cu^0^ is smaller than bulk fcc Cu^0^, indicating bulk and/or surface lattice defects associated with undercoordinated Cu atoms. This is in agreement with the in situ TEM results that suggested the formation of nano-sized Cu sheet fragments. Undercoordinated Cu sites formed during the CuO NS fragmentation and visualized in Fig. 3, typically exhibit strong chemisorption that has been associated with outstanding C-C coupling^23,25^.

### Mechanistic implications from DEMS-based catalytic rates and product onset potentials

A differential electrochemical mass spectrometry (DEMS) set-up equipped with an electrochemical capillary cell^12^ with milli-second time resolution enabled tracking kinetic product formation rates and yields under transient catalytic CO2RR. Thanks to its time resolution, the DEMS technique accurately captured the electrode potentials where product generation sets in (so-called *onset potentials*). Onset potentials are important mechanistic information that can be associated to DFT-computational mechanistic predictions. Reliable onset potential evolutions of a variety of CO2RR products have not been reported before.

**Fig. 5:**
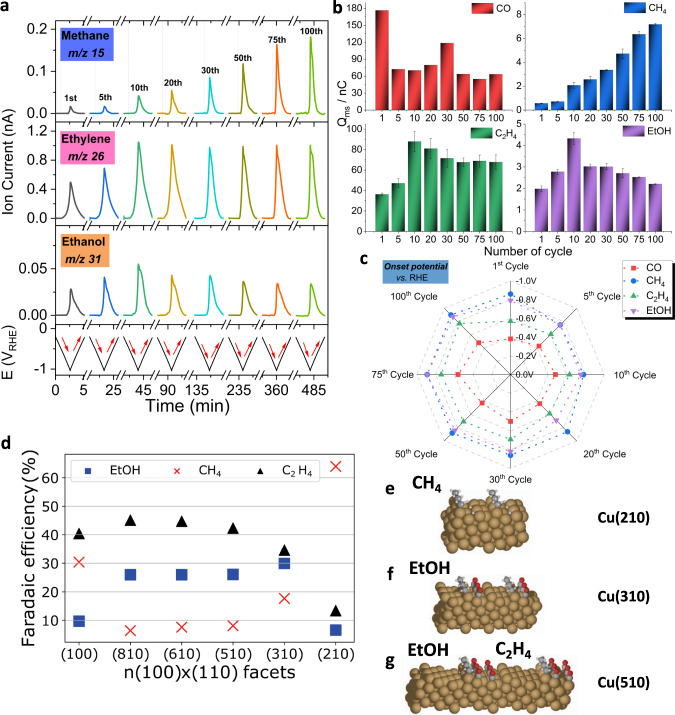
Products formation and onset potential shift along the electroreduction of CuO NS catalyst. **a** Operando differential electrochemical mass spectrometry (DEMS) sweep data obtained during CO2RR on CuO NS catalysts (supported on a flat 0.785 cm^2^ glassy carbon electrode, catalyst loading: 100 μg cm^−2^.) using CO_2_-saturated 0.1 M KHCO_3_ by continuous cyclic voltammetric scan at 5 mV s^−1^. **b** DEMS-derived mass charges for various products formed during the cathodic and anodic voltammetric sweep. The error bars are given as standard error of mean. **c** Spider plot shows the variations in the onset electrode potential of key products during CO2RR by continuous cyclic voltammetric scan at 5 mV s^−1^ (see Supplementary Table S2). Product molecules considered are: m/z = 28 CO, m/z = 15 corresponding to methane, m/z = 26 corresponding to ethylene, m/z = 31 corresponding to ethanol. **d** Faradaic efficiencies of CH_4_, EtOH and C_2_H_4_ on Cu(100) terraces of (from left to right) shrinking width and increasing density of Cu(110) steps. While Cu(100) shows little EtOH, EtOH increases with larger (110) step density and narrower (100) terrace width, peaking at Cu(310). The narrow (100) terraces on Cu(210) prevent EtOH formation in favor of CH_4_ (data from Ref. ^56^). **e**–**g** Illustration of *CH_3_ as common intermediate of CH_4_ and EtOH: Side views of the Cu(210), Cu(310) and Cu(510) single crystal facets. Cu(210) features exclusive *CH_3_ and *H adsorption toward CH_4_, while Cu(310) allows for C-C coupling between *CH_3_ and carbonaceous adsorbates on the (100) terraces toward EtOH. The wider (100) terraces of Cu(510) also enables C-C coupling among carbonaceous adsorbates to C_2_H_4_. Grey: C, white: H, red:O.

We performed a continuous 9-h DEMS measurement while cycling the potential between −0.2 V_RHE_ and −1.0 V_RHE_. The anodic turning potential was set deliberately as to prevent Cu from re-oxidation during the cycling. Each cycle took about 320 s. Figure 5a compares the transient mass ion currents of CH_4_, C_2_H_4_ and ethanol (EtOH) at m/z = 15, 26, and 31, respectively, as function of cycle number and time. C_2_H_4_ and EtOH production rates doubled during the first hour, peaked at the 10^th^ cycle (45 min), while CH_4_ production showed continues increase.

The integrated mass ion charges of CO, CH_4_, C_2_H_4_ and EtOH represent the product yields over cycle and time (Fig. 5b) and were correlated with the chemical state of the Cu catalyst. Parallel to the emerging defective metallic Cu^0^ catalyst, CH_4_, C_2_H_4_ and EtOH showed increasing rates and yields over the first 10 cylces (~45 min). Thereafter, C_2_H_4_ remained partly stable, while CH_4_ and EtOH showed opposite trends. This was in contrast to CO, the yield of which showed a complex response to the C_1_ and C_2_ pathways. The electrode onset potentials of these four key reaction products over 100 potential cycles are displayed in the spider/radar plot of Fig. 5c. This diagram represents—to our knowledge—the first such time-resolved evaluation of the evolution of product onset potentials. This experiment offers novel and noteworthy mechanistic implications and hypotheses. The vanishing oxidic character of CuO NS (Fig. 4c) coupled to sheet fragmentation and re-aggregation during the first hour (~10^th^ cycle) shifted the onset potentials of both C_2_H_4_ (green) and CO (red) from −0.57 ± 0.07 V_RHE_ and −0.38 ± 0.08 V_RHE_ to −0.63 ± 0.09 V_RHE_ and −0.48 ± 0.05V_RHE_, and after 9 h (100^th^ cycle) further to −0.77 ± 0.02 V_RHE_ and −0.48 ± 0.03 V_RHE_, respectively. Experimental onset potentials of Fig. 5c can be associated with the theoretical concept of limiting electrode potentials^51,52^ of the CO and C_2_H_4_ pathways, that is, the required minimal overpotentials before the last elementary kinetic step turns energetically downhill and the reaction proceeds^51^. The onset potentials of C_2_H_4_ and CO shifted concertedly, yet stayed apart by 150−250 mV. We attribute the shift to a decline in the number of active Cu surface sites. The varying offset represents the electrical energy (limiting overpotential) needed to kick-start the dimerization of *CO toward C_2_H_4_^53^ and corroborates their well-known mechanistic link.

More important are the mechanistic implications of the similar constant onset potentials of CH_4_ and EtOH (−0.8 V_RHE_ to −0.9 V_RHE_ over 9 h). There appears to exist a common elementary step that controls the onset potentials of both CH_4_ and EtOH. Against conventional mechanistic wisdom^53,54^, this suggests a much closer mechanistic link between CH_4_ and EtOH than previously thought^52,53^. We have previously observed an opposite correlation in CH_4_ and EtOH faradaic production^55^, by carrying out statistical analysis on the product formations on a series of Cu facets^56^. A careful observation was that, while the stepped Cu(210), i.e. (2(100)x(110)) surface is highly selective for CH_4_, Cu(n10), i.e., (*n*(100)x(110), *n* > 2) facets are highly EtOH selective^57^. Combining this analysis with the experimental observation here of onset potentials of CH_4_ and EtOH tracking each other—we put forward the new hypothesis that products with a common -CH_3_ group (CH_3_-H, CH_3_-COOH, CH_3_-CHO & CH_3_-CH_2_OH) could share a methyl intermediate. We speculate that 3 or more atom-wide (100) terraces at a (110) step enable the stabilization of an intermediate, that couple with *CH_3_ to EtOH. Figure 5e–g illustrate this new mechanistic concept. Reactive methyl *CH_3_ intermediates adsorbed at the (110) step while *CO preferably adsorbed on the (100) terraces. *CO dimerization on terraces forms C_2_H_4_ at lower overpotentials, while *CH_3_ require larger overpotential to become catalytically reactive at −0.8 V_RHE_. Given the terrace is wide enough, *CH_3_ combines with a carbonaceous adsorbate on Cu(100) to form EtOH. If the terrace becomes too small as on the (210) facet, it can no longer accommodate carbonaceous adsorbates. Now, *CH_3_ forms preferentially methane. This mechanism is directly confirmed by Cu single crystal FE data in Fig. 5d, however the concept of *CH_3_ as a common precursor for CH_4_ and EtOH has completely overlooked before, and hence is not incorporate in any of today’s mechanistic schemes. We note that this direct large-overpotential EtOH pathway is separate from the in-directly pathway, where acetaldehyde has been shown as a precursor for ethanol^58–60^, and we have discussed and shown that acetaldehyde can be reduced to EtOH^55^.

### CO2RR electrolysis using gas diffusion electrodes

**Fig. 6:**
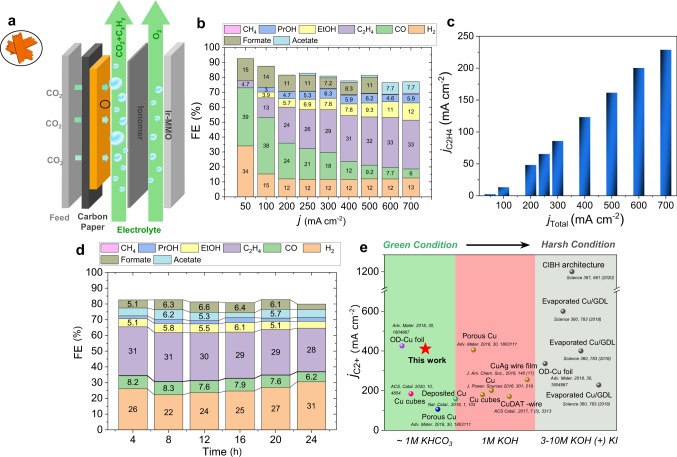
Electrochemical characterization in flow cell configuration. **a** Schematic representation of the flow cell electrolyzer. **b** Faradaic efficiencies as a function of applied geometric current density for CuO NS with catalyst loading of 1 mg cm^−2^ in 1 M KHCO_3_. **c** The partial current density of C_2_H_4_. **d** Stability test at a geometric current density of 300 mA cm^−2^, displaying the Faradaic efficiencies as a function of time. **e** Plots of C_2+_ partial current densities in a flow-cell system (compared with references)^4,43,46,63–68^.

To assess the potential of CO2RR on CuO NS under industrial pH neutral conditions (1 M KHCO_3_), we deposited the catalyst onto gas diffusion electrodes (GDE) separating CO_2_ and liquid catholyte flows in single cell electrolyzer set ups (Fig. 6a). Constant current densities between 50 and 700 mA cm^−2^ were applied for 2 h.

Figure 6b shows the product FEs versus currents at neutral pH. The initial large H_2_ FE at 50 mA cm^–^^2^ is owed to relatively low electrode overpotentials at the metallic Cu^0^ sheet fragments. With increasing currents, the Cu catalyst developed its excellent CO_2_RR FE of 65% at 700 mA cm^−2^, with mere 13% H_2_. As the C_2_H_4_ FE reached 33% at neutral pH, the partial C_2_H_4_ current maxed at 229 mA cm^−2^ (Fig. 6c). Previously unachieved is the C_2+_ product partial current density of 410 mA cm^−2^ (Supplementary Fig. S26). The fact that CH_4_ formation is essentially suppressed at all currents (e.g., 0.58% at 700 mA cm^−2^) making C_2_H_4_ the sole gaseous hydrocarbon product, merits further attention. Stability tests at 300 mA cm^−2^ for 24 h (Fig. 6d) revealed only negligible FE_C2H4_ drops over 20 h. Consistent with in situ TEM data, agglomeration resulted in long-term dendritic morphologies (Supplementary Figs. S27 and S28). To put the excellent performance of CuO NS precursors and its morphological derivatives in perspective, Fig. 6e compares the C_2_H_4_ partial currents under comparable conditions. Our CuO NS meet and exceed today’s top performing Cu catalysts under neutral condition in electrolyzers.

## Discussion

We have studied the synthesis and CO_2_ electrocatalysis of a (001)-oriented CuO nanosheet catalyst. Evaluated in electrolyzers under industrially relevant pH neutral conditions, the CuO NS catalysts featured unprecedented performance metrics for C_2_H_4_ and C_2+_. In situ and ex situ TEM studies revealed that over 10–20 min under potential control the CuO NS fractured and—presumably limited by diffusion of Cu species—aggregated into branched, dendritic Cu structures that represented the final stable catalyst morphology under flow conditions. Operando XAS studies confirmed the chemical reduction of CuO and concomitant formation of disordered and coordinatively under-saturated Cu^0^ over about 2 h under reductive CO2RR conditions. These undercoordinated sites are held responsible for the high catalytic CO_2_-to-C_2+_ product reaction rates. Time-resolved DEMS provided access to the evolution of product onset potentials. The concerted shift in onset potentials of CO and C_2_H_4_ evidenced their close mechanistic link. Those of CH_4_ and EtOH, by contrast, remained unaffected and were nearly identical. Counter to our current mechanistic understanding, this points to the existence of a common mechanistic intermediate, likely *CH_3_, feeding into competing pathways toward EtOH and CH_4_, confirming previously overlooked data trends on metallic stepped Cu(n10) single crystal facets. Based on this, Tetrahexahedrally shaped^61,62^ particles exposing stepped (310) facets are predicted be optimal nanocatalysts to support the EtOH-selective pathway.

## Methods

### Synthesis

Nanosized CuO catalyst with sheet-like morphology was synthesized by a surfactant-free wet-chemistry route. No purification was performed for chemicals before use. CuO nanosheets were obtained by thermal decomposition of pre-synthesized Cu(OH)_2_ intermediate. To prepare Cu(OH)_2_ intermediate, 100 mg Cu(SO_4_)_2_ was dissolved in 4 mL DI-water, 2.5 mL 1 M KOH solution was then added dropwise into the solution. After 15-min stirring, 1 mL ammonium hydroxide was added. The resulting homogeneous light blue solution was then transferred to a glass pressure vessel. The sealed vessel was then heated from room temperature to 80 °C in 30 min and stayed at 80 °C for 12 h before it was cooled to room temperature. The products were precipitated by ethanol, separated via centrifugation at 12,649.63 g and further purified twice by ethanol and water. Afterwards they were freeze dried and stored as powders under inert atmosphere until use.

### Electron microscopy (TEM, HRTEM and SAED)

TEM and SAED were performed using a FEI Tecnai G^2^ Microscope 20 S-Twin with a LaB6-cathode at 200 kV accelerating voltage (ZELMI Centrum, Technical University Berlin). The samples were dispersed in ethanol, ultrasonicated and drop-dried onto Cu-grids with a holey carbon film. Analysis was done using software from ImageJ. The HR-TEM was conducted with FEI TITAN 80–300n with high brightness FEG (ZELMI Centrum, Technical University Berlin).

### Electrochemical Measurements in H-cell

The electrochemical tests were firstly carried out in a custom-made two-compartment cell (H-cell), separated by an anion exchange membrane (Selemion AMV, AGC Engineering Co., LTD). All the glassware accessories used in this study were first cleaned by soaking in a “nochromix” bath and afterward in concentrated HNO_3_ for at least 12 h, rinsed and sonicated with ultrapure water several times. We have worked with a constant concentration of 0.1 M CO_2_-saturated KHCO_3_ solution (Honeywell). The working/counter compartment was filled with 40 mL of electrolyte respectively. Before and during the electrochemical reaction the working compartment was purged continuously with CO_2_ (30 sccm in total) from the bottom of the cell and the gas atmosphere was controlled with an in situ mass flow controller. Polished glassy carbon or carbon paper are used as working electrodes and measured with a Biologic SP 300 potentiostat. A platinum mesh 100 (Sigma-Aldrich 99.9%) was used as counter electrode (CE) and a leak-free Ag/AgCl electrode as reference electrode (Multi Channel Systems MCS GmbH). Every measurement was started with linear sweep voltammetry (LSV), performed with a scan rate of −5 mV/s starting at E = + 0.05 V_RHE_ and ending at the working potential (between −0.78 V_RHE_ and −1.0 V_RHE_) followed by a chronoamperometric step for a certain time. All reported potentials are corrected for Ohmic drop determined by electrochemical impedance spectroscopy (EIS). EC-Lab software was used to automatically correct 50% of the Ohmic drop, the remaining 50% was corrected manually. For each measurement, fresh electrolyte was used to ensure that adsorbates from previous experiments did not influence the result.

### In situ transmission electron microscopy

Poseidon Select electrochemical cell holder (Protochips, in situ TEM E-chip cell) was used to load the samples into the microscope and maintain the liquid environment for the experiment. The electrochemical cell was made from commercially available electrochemistry chips (Protochips) consisting of an O-ring, a bottom chip, and a top E-chip with 3-electrodes imprinted on it, as shown in Supplementary Figure S13. The 3 electrodes on the top E-chip are made from Pt (both the reference and counter electrode), and glassy carbon (the working electrode). Both top and bottom chips are delivered with a protective photoresist coating to prevent damage to the SiN membrane. Acetone and methanol were used to remove the protective photoresist coating. The CuO NS catalyst were dispersed in ethanol and drop-casted onto the center of the top chip, plasma cleaning for 20 s improves the hydrophilicity of the chips before use. After assembling, liquid solution was delivered by an external Hamilton syringe pump through the microfluidic tubing into the tip of the Poseidon Select TEM holder with a flow rate of 30 μL/h^−1^. The electrochemistry measurements were performed with a floating potentiostat (Gamry Reference 600 + ). More experimental details are presented in the supplementary information.

### Operando X-ray absorption spectroscopy (XAS)

Operando X-ray absorption spectroscopy was measured at the Cu K-edges at the BESSY-II synchrotron facility at KMC-3 beamline (Helmholtz-Zentrum Berlin, Germany). CuO NS catalysts were prepared on 2×2.5 cm glassy carbon sheets (250 µm thickness, Sigradur K) and mounted in an in-house made electrochemical Teflon cell. Operando spectra of CuO NS samples were collected in fluorescence geometry from the backside of the glassy carbon electrode. We used a 13 element Si-drift energy resolving detector (RaySpec) which we equipped with an Al-shielding having a 25×25 mm Ni foil (0.00125 mm, 99.999%, Goodfellow) in front to suppress scattered light. The electrochemical cell was controlled by a SP-300 potentiostat (Biologic). We used a Ag/AgCl reference electrode and a Pt coil as counter electrode. The CuO area exposed to the electrode was 1.96 cm². The 0.1 M KHCO_3_ electrolyte was purged throughout the experiment with ≈20 ml/min. All potentials were compensated for 85% Ohmic drop (R ≈ 40 Ω).

### Operando differential electrochemical mass spectrometry (DEMS)

Operando Differential electrochemical mass spectrometry (DEMS) was done using a custom-made TU Berlin electrochemical capillary DEMS flow cell. DEMS capillary flow cell is characterized by having a well-defined electrolyte profile flow over the working electrode. The reaction products are transported into the interface liquid vacuum throughout a 0.15 mm glass capillary. The collection of high concentrated aliquot near catalyst surface and enhanced liquid-vacuum interface allows a fast and at high-intensity detection of gaseous products. The high performance with DEMS capillary flow cell results in the distributed flow over the hydrophobic membrane compartment also known as the cyclonic flow. The interface liquid/vacuum promoted by PTFE hydrophobic membrane with a pore size of 20 and thickness of 50 *μ*m (Cobetter®, Cat. No. PF-002HS) are commercially available at Hangzhou Cobetter Filtration Equipment Co., Ltd. The reaction products after been vaporized into the vacuum chamber from the flow cell were detected using a PrismaTM quadrupole mass spectrometer (QMS 200, Pfeiffer-Vacuum). The vacuum chamber was composed of two turbomolecular pumps (HiPace 80) that perform an ultimate pressure 10^−6^ mbar at MS detectors. Each turbomolecular pump has an independent baking system helped a by membrane and oil pump (coupled with a molecular sieves oil trap).

### CO_2_ reduction in flow cell electrolyzer

The flow cell electrolyzer is used for measuring the samples at high current densities, which were performed in a commercial cell supplied by ElectroCell. In all flow-cell experiments a commercial Ir-MMO plate (ElectroCell) was used as anode. The catalyst-inks were spray-coated on the microporous layer (MPL) of a Freudenberg C2 gas diffusion layer (GDL) on an area of 3 cm^2^ to achieve a catalyst loading of 1 mg cm^−2^. Nafion (Sigma-Aldrich, 5 wt% resin solution) was used as binder and for ionic conductivity of the catalyst-layer. 1 M KHCO_3_ (500 mL, Sigma-Aldrich, BioUltra, ≥99.5%) was used as anolyte and catholyte, which were separated by an anion exchange membrane (Selemion AMV, AGC Engineering Co., LTD.). Both electrolytes were cycled through each respective compartment at 100 mL min^−1^ by using a peristaltic pump (PMP Ecoline, Cole-Parmer). The CO_2_ (4.5 N) was supplied at a rate of 50 mL min^−1^ to the cathode and was flown from the back of the GDL through the catalyst layer. Measurements were performed galvanostatically for 2 h at each respective current during the catalytic tests, changing the current from low to high values. Each galvanostatic step was followed by a Potentiostatic Electrochemical Impedance Spectroscopy (PEIS) measurement to account for the ohmic drop in the calculation of RHE potentials.

## Supplementary information

Supplementary Information

Description of Additional Supplementary Files

Supplementary Movie 1

Supplementary Movie 2

Supplementary Movie 3

Supplementary Movie 4

Supplementary Movie 5

Supplementary Movie 6

## Data Availability

All data that support the findings of this study are present in the paper and the supplementary materials, and additional data are available from the corresponding author upon reasonable request. The Supplementary Information contains descriptions of methods, discussions on physicochemical characterization of CuO NS, in situ TEM, operando XAS, DEMS and flow cell test. The XAS data for Cu, and Cu_2_O reference materials were taken from ref. ^20^.
