# Supplementary material for: Morphology and mechanism of highly selective Cu(II) oxide nanosheet catalysts for carbon dioxide electroreduction
Source: Nat Commun. 2021 Feb 4;12:794. doi: 10.1038/s41467-021-20961-7 (PMC7862240; doi:10.1038/s41467-021-20961-7)
Supplement: Supplementary file 1 — Supplementary Information [file 41467_2021_20961_MOESM1_ESM.pdf]

Supporting Information

Morphology and Mechanism of Highly Selective Cu(II) Oxide Nanosheet Catalysts for Carbon Dioxide Electroreduction

Xingli Wang[1], Katharina Klingan[2], Malte Klingenhof[1], Tim Möller[1], Jorge Ferreira de Araújo[1], Isaac Martens[3], Alexander Bagger[4], Shan Jiang[2], Jan Rossmeisl[4], Holger Dau[2], and Peter Strasser[1*]

[1] Department of Chemistry, Chemical Engineering Division, Technical University Berlin, Straße des 17. June 124, 10623, Berlin, Germany

[2] Department of Physics, Free University of Berlin, Arnimallee 14, 14195 Berlin, Germany, Germany

[3]European Synchrotron Radiation Facility (ESRF), 38000 Grenoble, France

[4]Department of Chemistry, University of Copenhagen 2100, Denmark

Contacts:

pstrasser@tu-berlin.de

This Supplementary Information file includes:

Experimental Details

Supplementary Figures S1 to S28

Supplementary Tables S1 to S2

Supplementary Movie 1 to 6 (Separate files)

References

Supplementary Methods

Scanning electron microscopy (SEM)

The SEM was measured using a JEOL 7401F instrument at an acceleration voltage of 10 kV and a working distance of approximately 4.1 mm. Image J was employed to analyze the SEM images.

Synchrotron Wide-Angle X-Ray Scattering (WAXS) Measurements

Synchrotron WAXS measurements were recorded at beamline ID31 at the European Synchrotron Radiation Facility (ESRF) in Grenoble, France at an energy of ~ 70 keV using a large area detector (Pilatus 3X CdTe 2M). The sample was prepared and measured directly on the glassy carbon electrode.

Working Electrode Preparation

The working electrodes were drop-coated with certain amount of catalyst ink. The ink for CuO nanosheets (CuO NS) consists of 2 mg electrocatalyst powder; 0.8 mL of ultrapure water; 0.15 mL isopropanol; 50 μL of 5 wt. % Nafion solution. CuO NS ink was used for drop-coating onto glassy carbon plates for H-cell measurements, and glassy carbon cylinders for DEMS measurements. The geometric catalyst loading was kept as 100 ug/cm^2.

The working electrodes with carbon paper electrodes in H-cell configuration for the morphology evolution experiment were prepared with catalyst ink containing 2 mg electrocatalyst powder; 0.2 mL of ultrapure water and 0.8 mL isopropanol. The geometric catalyst loading was kept as 100 ug/cm^2 as well.

In flow cell configuration, the catalyst ink (a solution of 6 mg catalyst, a certain amount of 5 % Nafion, isopropanol and ultrapure water) was spray-coated on the microporous layer (MPL) of a Freudenberg C2 gas diffusion layer (GDL). A geometric area of 3 cm^2 was coated to achieve a final catalyst loading of ~1 mg cm^{-2}. Additionally, 30 wt% of Nafion was used in the catalyst ink as a binder and for ionic conductivity of the catalyst layer.

in situ Transmission Electron Microscopy

A Poseidon Select electrochemical cell holder (Protochips, *in situ* TEM E-chip cell) was used to load the samples into the microscope and maintain the liquid environment for the experiment. After assembling, water was delivered by an external Hamilton syringe pump through the microfluidic tubing into the tip of the Poseidon Select TEM holder with a flow rate of 30 μL/h^{-1}. The liquid flow rate was carefully controlled in case of bending SiN membranes, which may cause problems for the later imaging step, due to the increased liquid thickness. The flow rate was kept at 30 μL h^{-1} during a vacuum check before

insertion into the microscope column, and during imaging. Microscopy was performed using a FEI TECNAI G ^{2} 20 S-TWIN microscope with a GATAN MS794 P CCD-detector at 200 kV. The images were acquired by image sequences every 2/2.5 s, stacking as movies with 5 frames/s by Fiji.

The electrochemistry measurements were performed with a floating potentiostat (Gamry Reference 600+). OCP measurement was firstly performed in milli-Q water with a flow rate of 30 μL h^{-1}. Real-time imaging of the CuO NS catalyst under OCP is shown in Supplementary Movie 1. Water was then replaced by 0.1M Na_2HPO_4/NaH_2PO_4 (pH=6.9) (Sigma Aldrich with milli-Q water) buffer electrolyte feed, Electrochemical Impedance Spectroscopy (EIS) measurement was performed for determination of the Ohmic resistance. Real-time imaging of the CuO NS catalyst under the solution change (water against buffer) is shown in Supplementary Movie 2 and 3. The electrochemical measurement was started with a linear voltammetric sweep (LSV) after a 10-second OCP test, performed with a scan rate of -50 mV/s between OCP and -0.84 V_{RHE} followed by a chronoamperometric (CA) step at -0.84 V_{RHE} for a certain time. Real-time imaging of the CuO NS catalyst under first LSV+CA is shown in Supplementary Movie 4. The second LSV and CA was performed on a more negative potential, -1.23 V_{RHE}, the real time imaging is shown in Supplementary Movie 5. The third LSV and CA was performed on -1.73 V_{RHE}, the real time imaging is shown in Supplementary Movie 6. To control affects arising from beam damage and liquid layer influence, experiments are also taken with the identical location (IL) TEM E-chip cell identical location (IL) TEM E-chip cell using the same procedure with same chip design. The chips used in the IL E-chip cell were re-checked in microscopy with *in situ* TEM E-chip cell (Poseidon holder) in dry state after rinsing.

After use, a cleaning step was carefully performed. All lines were purged with water overnight and rinsed by ethanol. Dry air was then used to purge the lines and flush the holder tip.

The potential setting in the floating potentiostat was according to:

$$E_{Apply} = E_{RHE} + E_{adj} \quad (1)$$

$$E_{adj} = E_{0-Pt} + 0.059 * pH \quad [E_{0-Pt} = -1.18\,V] \quad (2)$$

Since the current was quite low in the IL E-chip cell and *in situ* TEM E-chip cell, IR correction was not considered.

Notably, as potassium ions are harmful to the SiN membrane, instead of CO_2-saturated 0.1M $KHCO_3$ an electrolyte solution of 0.1M Na_2HPO_4/NaH_2PO_4 (pH=6.9) was used in the *in situ* TEM E-chip micro reactor to maintain a similar pH value and avoid membrane breakage. Gas bubbles inside the cell disrupt

the electrochemical measurement, thus, controlling bubble generation is necessary. The control experiment with N_2-saturated 0.1M Na_2HPO_4/NaH_2PO_4 (pH=6.9) electrolyte was also performed in H-cell geometry to exclude the effect on CO_2-reactant (see Supplementary Figure S21).

operando X-ray absorption spectroscopy (XAS)

The reduction behavior and local atomic information of CuO NS during CO2RR were followed by *operando* XAS at the Cu K-edge. Several materials were used for collection of reference spectra: CuO (nanopowder, particle size < 50 nm, surface area 29 m²/g, Sigma Aldrich), $Cu(OH)_2$ (self-synthesized, described in main part under the section “Synthesis”), Cu, and Cu_2O was taken from ref. https://doi.org/10.1002/cssc.201801582. Reference powders CuO and $Cu(OH)_2$ were mixed and ground with boron nitride, and measured at 20 ℃ in absorption mode at the Cu K-edge with ionization chambers before and after the samples. Energy calibration was done by simultaneously measuring a Cu foil (0.001 mm, 99.999%, Goodfellow) and shifting the energy axis to the first fitted maximum of the derivative of the absorption of the Cu foil. The metal foil reference was measured in absorption mode as well.

CuO NS catalysts were prepared on 2x2.5 cm glassy carbon sheets (250 µm thickness, Sigradur K) and mounted in an in-house made electrochemical Teflon cell. *Operando* spectra of CuO NS samples were collected in fluorescence geometry from the backside of the glassy carbon electrode. We used a 13 element Si-drift energy resolving detector (RaySpec). The detector was equipped with Al-shielding and a 25x25 mm Ni foil (0.00125 mm, 99.999%, Goodfellow) to suppress scattered light.

The electrochemical cell was controlled by an SP-300 potentiostat (Biologic). We used an Ag/AgCl reference electrode and a Pt coil as a counter electrode. The CuO NS area exposed to the electrode was 1.96 cm². The 0.1 M $KHCO_3$ electrolyte was purged throughout the experiment at ≈ 20 mL/min^{-1}. All potentials were compensated for 85% ohmic drop (R≈40 Ω).

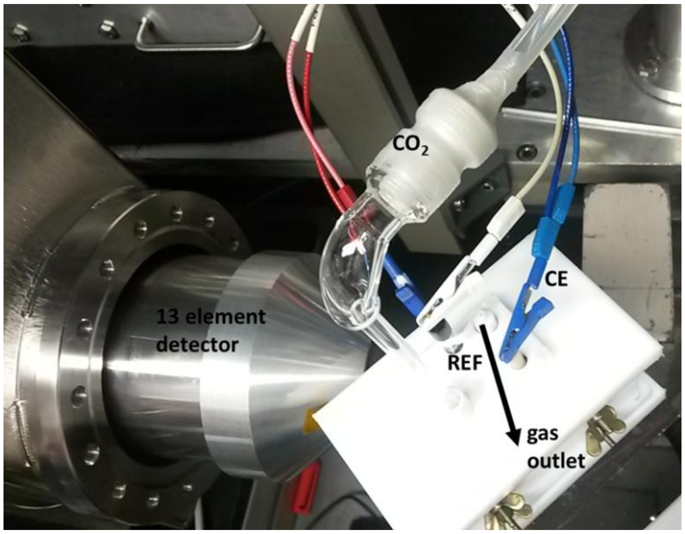

Supplementary Figure S1. *Photograph of operando XAS set-up at beamline KMC-3 at Helmholtz-Zentrum in Berlin (Bessy).*

We collected EXAFS spectra to **k**=12.2 with 6 min data acquisition time. The experimental protocol was as follows:

1. Spectrum of dry film
2. Spectrum of film at OCP
3. LSV with 5 mV/s from -0.02 to -0.84 V_{RHE}
4. Spectra taken between 0 min to 130 min every 10 min while -0.84 V_{RHE} was applied

During step 4, the beam shutter was closed after each spectrum (after 6 min data acquisition time) and three different sample spots were used to protect the sample from radiation damage. The experimental protocol was repeated twice, and the three individual data sets have been averaged accordingly. Energy calibration was done by measuring Cu foil and shifting the energy axis to the first inflection point of the fluorescence spectrum of Cu foil, before and after the experimental protocol.

EXAFS simulations

The extracted spectra were weighted by $\mathbf{k}^3$ and simulated in **k**-space. All EXAFS simulations were performed using in-house software (SimXLite) after calculation of phase functions with the FEFF program (version 8.4, self-consistent field option activated). Phase functions were calculated using geometries from open-access cif files of Cu, CuO, $Cu(OH)_2$, and the extracted cif files of CuO NS and $Cu(OH)_2$ from experimental XRD data. As usual, the EXAFS phase functions did not depend strongly on the details of the used model. Cosine windows covering 10% at the low-**k** and high-**k** side of the spectra

were applied before calculation of the Fourier transforms. An amplitude reduction factor (S_0^2) of 0.8 was used. The data range used in the simulation was 34.3-461.1 eV (3-11 Å^{-1}). The Debye-Waller parameters for all shells were fixed to avoid overparameterization, and to emphasize the changes in coordination number of the samples. The fixed Debye-Waller parameters have been chosen as followed: CuO powder reference, Cu metal reference, and CuO NS (at OCP) spectra have been simulated with fixed coordination numbers. The so obtained Debye-Waller parameters have been used for the simulation of the respective shells. The EXAFS simulation was optimized by a minimization of the error sum obtained by summation of the squared deviations between measured and simulated values (least-squares fit). The fit was performed using the Levenberg-Marquardt method with numerical derivatives. The error ranges of the fit parameters were estimated from the covariance matrix of the fit and indicate the 68 % confidence intervals of the corresponding fit parameters. The fit error was calculated as in reference[1]. For calculation of the Fourier-filtered error (described in reference[2]), the range from 1 to 6.5 Å on the reduced distance scale was used.

operando Differential electrochemical mass spectrometry (DEMS)

The onset potential is defined as the potential where the ion current signal reaches 1% from the highest detected signal of a correspondent product between all gas feeding systems during a cathodic voltammetric scan of 5 mV/s. To compensate for data noise variation during the determination of the potential onset, the onset values were taken from the average of a set consisting of consecutive neighbor cycles.

MS sensor (PrismaPlus QMS 220, Pfeiffer-Vacuum with tungsten filament) was calibrated prior to DEMS measurement. The ion source was set with manufacturer recommended filament current emission of 1000 µA, and electron energy was kept at 70 eV as a standard for molecule fragmentation. The peak intensity and peak shape were adjusted at ion source settings, such as extraction, field axis and focus.

To increase the detection sensitivity and simultaneously preserve the signal linearity, the SEM potential calibration was carried out by keeping signal intensity three order magnitude higher than Faraday cup detector. The setting of SEM detector is typical at around 1000 V. The preservation of linearity and most importantly the cracking (fragmentation) patterns of different gases is essential for an accurate deconvolution of mass signals during DEMS analysis. The DEMS instrument technology as well the electrochemical capillary flow cell used in this work are commercially available at www.liquidloop.eu.

Electrochemical Method

RHE Potential:

$$E_{RHE} = E_{Ref} + E_{Ag/AgCl} + 0.059 * pH + R * I \qquad (3)$$

E_{RHE}: RHE potential / V

E_{Ref}: Applied WE potential against the Ag/AgCl reference electrode / V

$E_{Ag/AgCl}$: Potential of the reference electrode measured against NHE (0.21 V) / V

pH: pH-value of the electrolyte

R: Ohmic resistance between working and reference electrode / Ω

Chromatographic Product Analysis

Gas samples were analyzed with a gas chromatograph (Shimadzu GC 2014) equipped with a thermal conductivity detector (TCD) and a flame ionization detector (FID). Argon (Air liquid 5.0) was employed as the carrier gas. The gaseous compounds H_2, N_2, O_2, CH_4 and CO were separated in a molecular sieve column (Alltech, part no. 57732, 1.65 m × 1/8 in., molecular sieve 13X, 60/80 mesh) while C_2-C_3 hydrocarbons and CO_2 were resolved in a HayeSep column (Alltech, part no. 14487, 3.5 m × 1/8 in., HayeSep D, 80/100 mesh).

The production rate towards the gas products was calculated taking into account the concentration obtained for the gas chromatography analysis and the feed flow (30 sccm) according to equation 4:

$$\dot{n} = \frac{\dot{V} * C}{A * V_M} \qquad (4)$$

$\dot{n}$: Generation rate of the product / mol s^{-1} cm^{-2}

$\dot{V}$: CO_2 gas flow rate / L s^{-1}

C: Volume/Molar fraction of detected product by GC (assuming ideal gas) / Vol%

A: Geometric area of the electrode / cm^{-2}

V_M: Gas Molar Volume / 22.4 L mol^{-1}

The measured molar production rate was used to calculate the Faradaic efficiency and the average current density measured during the last minute of reaction:

$$FE = \frac{\dot{n} * z * F}{j_{total}} * 100\% \qquad (5)$$

$\dot{n}$: Molar production rate of product per unit area / mol s^{-1} cm^{-2}

FE: Faradaic Efficiency of the product / %

z: number of transferred electrons per mol of product

F: Faraday constant / C mol^{-1}

j_{total}: Total current density during CO_2 bulk electrolysis / mA cm^{-2}

2 mL of the electrolyte after reaction was analyzed by high performance liquid chromatograph (HPLC Agilent 1200, Zimmer Chromatography® Column, RID detector) to measure formic acid concentration and analyzed by liquid injection gas chromatography (Shimadzu GC 2010 plus, Fused-Silica-Capillary Column, REF 723060.30, FID Detector) for alcohol products. Therefore, the production rate was calculated considering the total charge transfer, according to equation 6:

$$\dot{n}_x = \frac{V * \Delta C_x}{A * \Delta t} \qquad (6)$$

$\dot{n}_x$: Generation rate of the product x / nmol s^{-1} cm^{-2}

V: Volume of the electrolyte / ml

ΔC_x: Accumulated concentration of the product x detected by HPLC or liquid GC / mmol L^{-1}

A: Geometric area of the electrode / cm^{-2}

Δt: Reaction time at const. current or potential / s

Faradaic efficiency of liquid products was calculated by equation 7:

$$FE_x = \frac{V * \Delta C_x * z_x}{\Delta Q} * 100\% \qquad (7)$$

FE_x: Faradaic Efficiency of the product x / %

V: Volume of the electrolyte / ml

ΔC_x: Accumulated concentration of product x detected by HPLC or liquid GC / mmol L^{-1}

z_x: electrons transferred for reduction to product x

ΔQ: Total charge transfer during the electrolysis at const. potential or current / C

The partial current density is given by,

$$j_x = \frac{FE_x * j_{total}}{100} \qquad (8)$$

j_x: Partial current density / mA*cm^{-2}

FE_x: Faradaic Efficiency of the product x / %

j_{total}: Total current density / mA*cm^{-2}

Physical and Chemical Characterizations

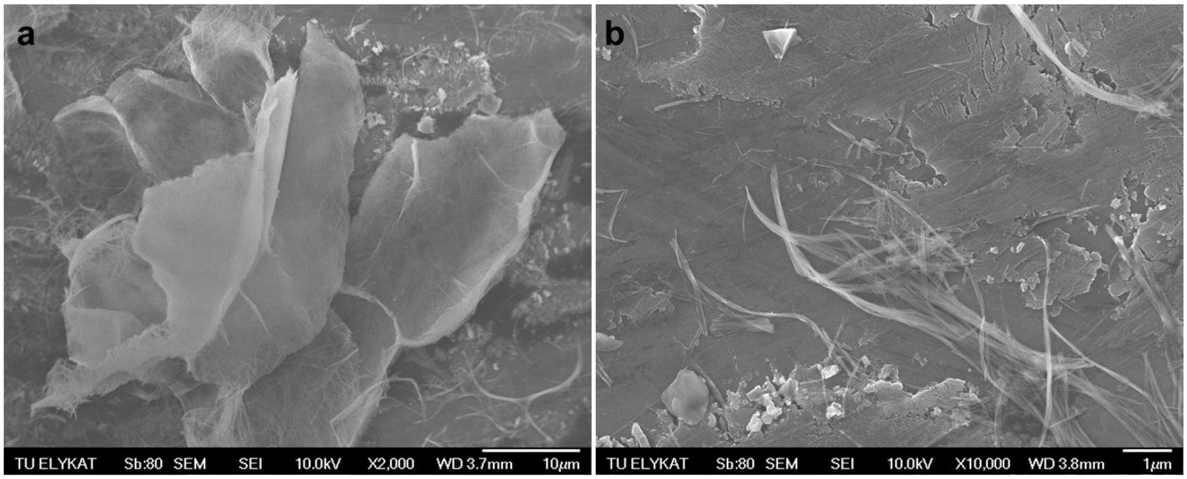

Supplementary Figure S2. *Ex situ SEM images of $Cu(OH)_2$ intermediate formed during synthesis process.*

The transformation process of $Cu(OH)_2$ to CuO was suggested by Cudennec and Lecert[3] as:

$$Cu(OH)_2\ (s) \rightarrow CuO\ (s) + H_2O \qquad (9)$$

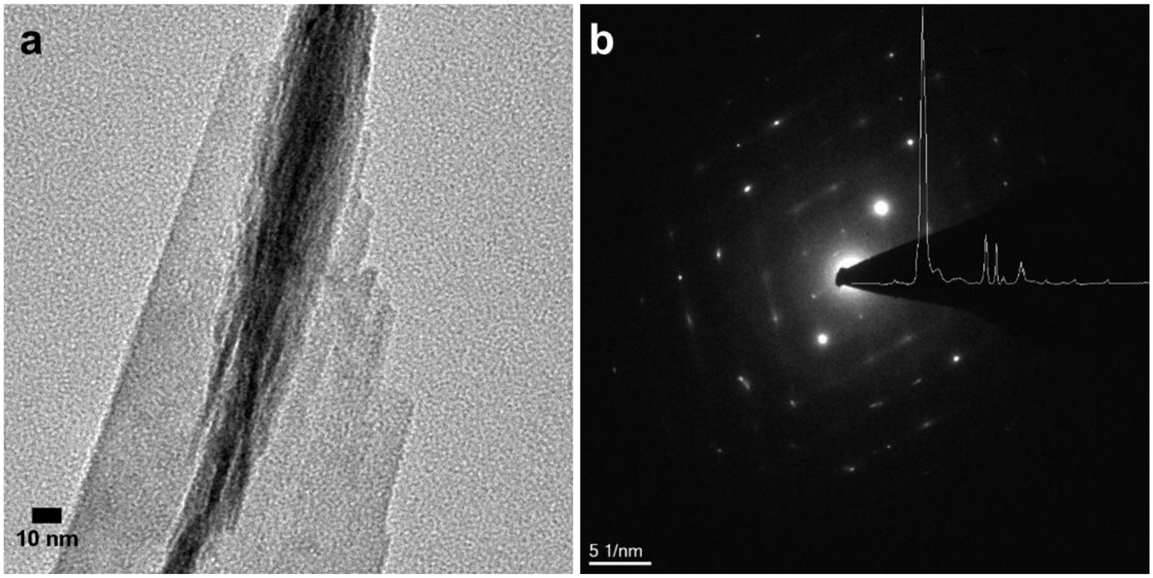

Supplementary Figure S3. *a) Cross-section TEM images of as-prepared CuO NS and b) corresponding intensity of SAED intensity.*

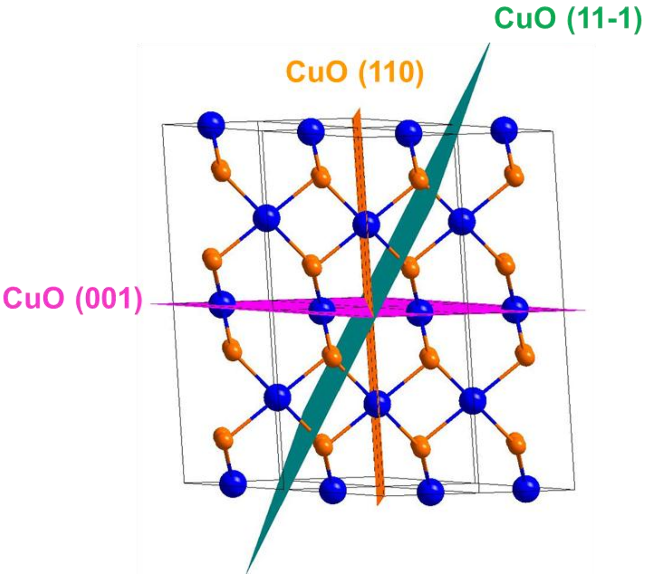

Supplementary Figure S4. *Crystal structure of monoclinic CuO with indexing plane.*

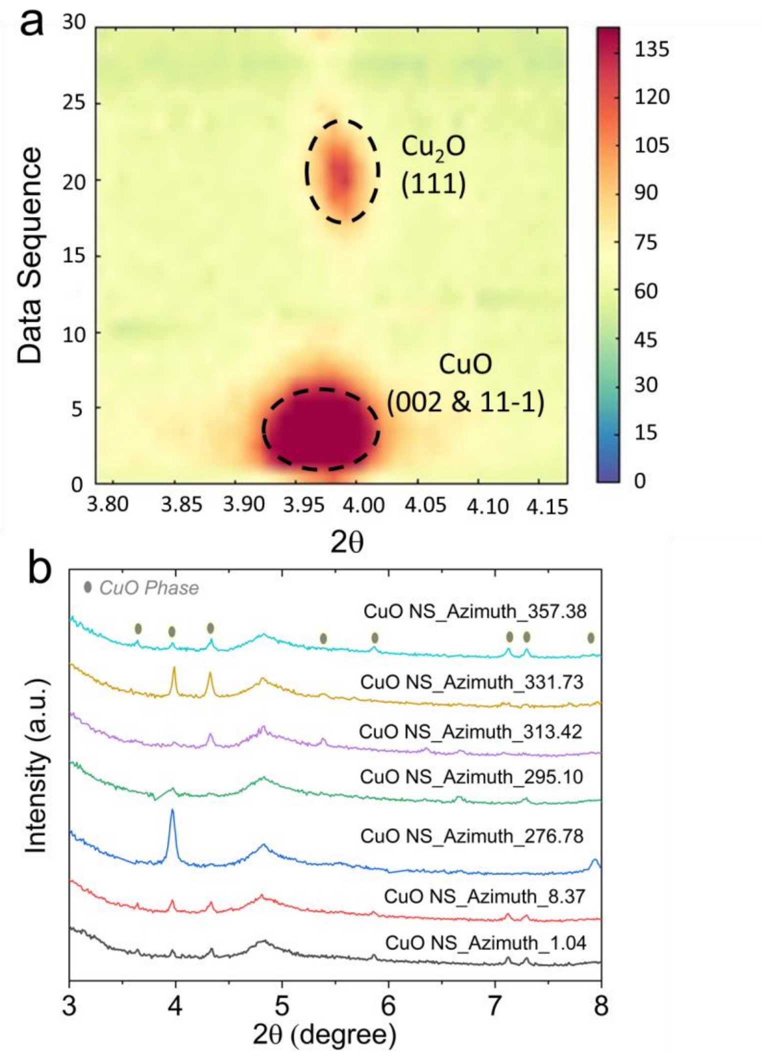

Supplementary Figure S5.GI-WAXS: *a) Partial contour plot of CuO NS. b) Azimuthally integrated line profiles of as-prepared CuO NS.*

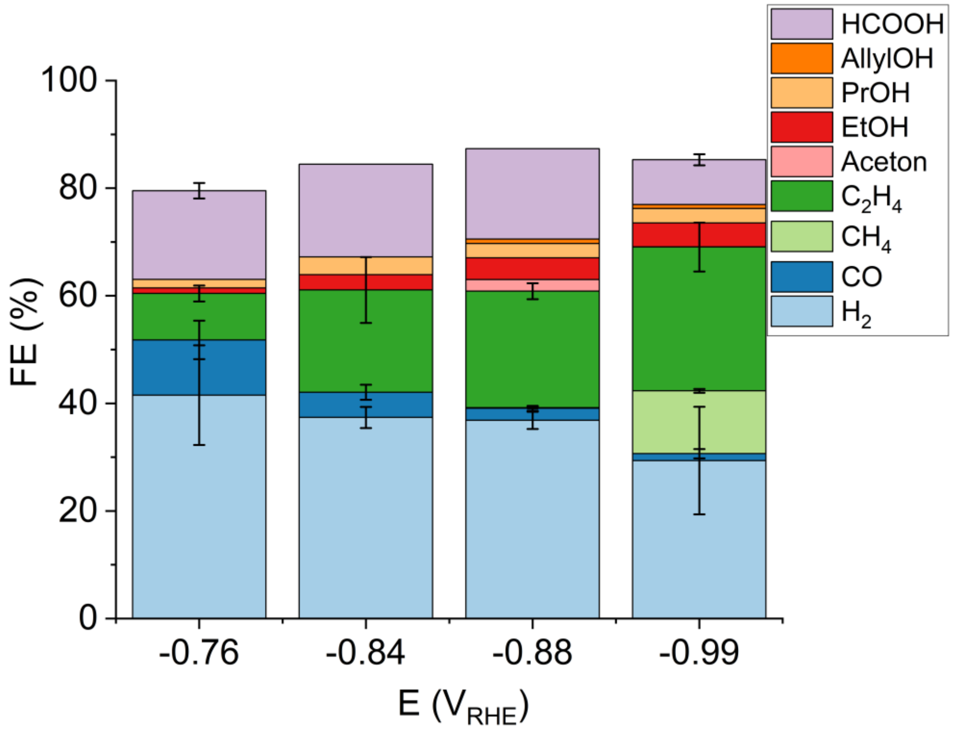

Supplementary Figure S6. Gaseous and liquid products analysis. *Faradaic efficiencies of detectable main products distribution after 1-h CO2RR in H-cell design. The errors are given as SE of mean from different experiments.*

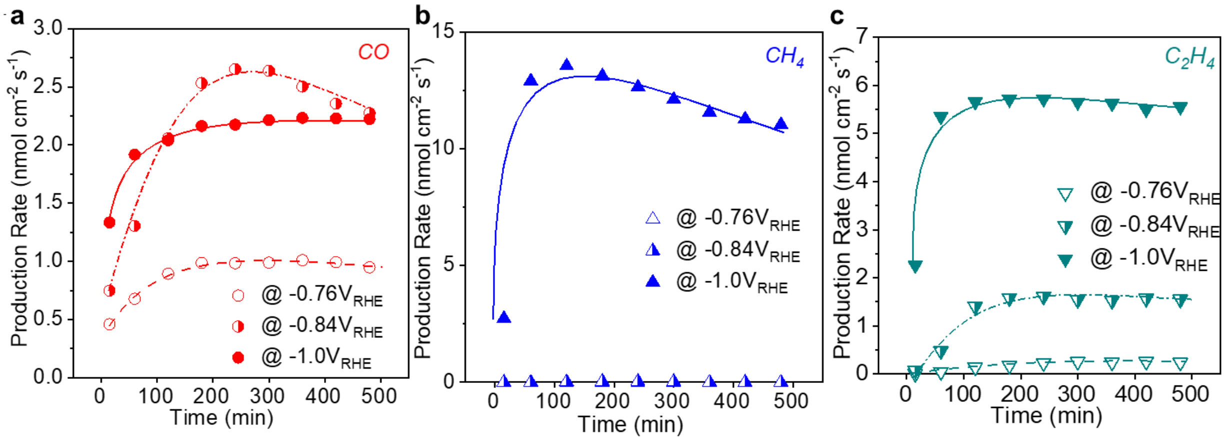

Supplementary Figure S7. Potential-dependent stability tests up to ca 9 h. *Time-dependent absolute product formation rates on a) CO, b) CH_4 and c) C_2H_4 with various overpotentials.*

Morphology evolution determination after short-term reaction:

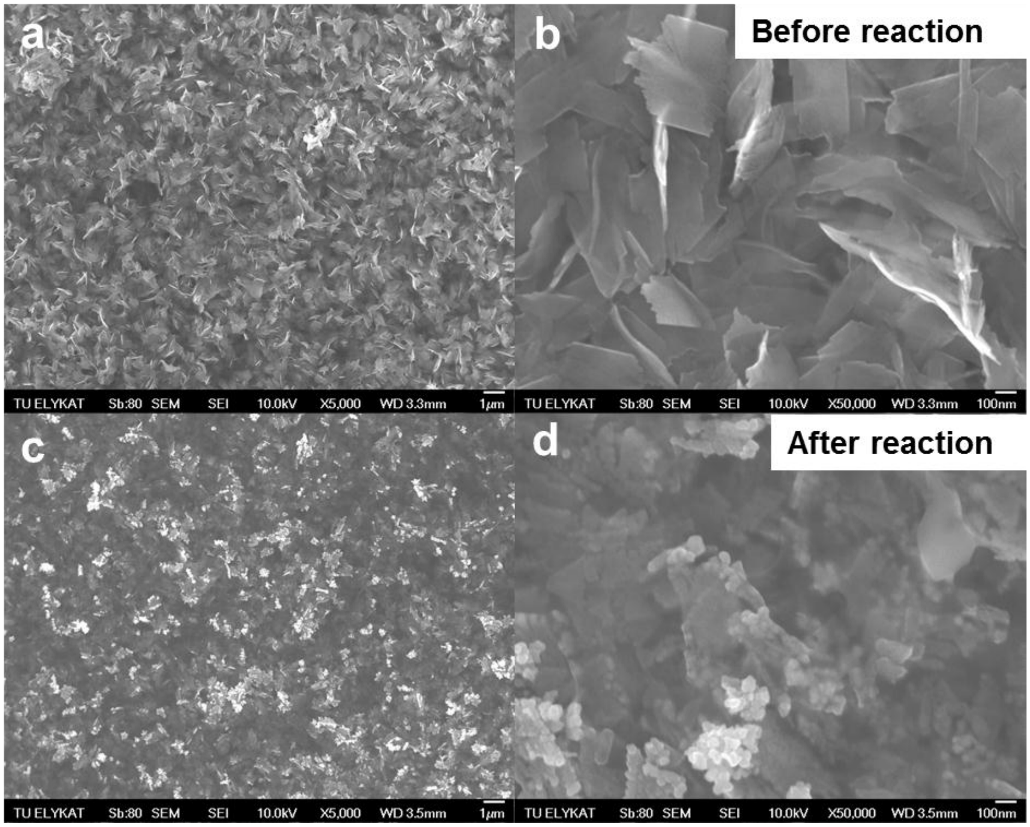

Supplementary Figure S8. Morphological changes after short-term reaction in H-cell observed by ex situ SEM. *of CuO NS in CO_2-saturated 0.1M $KHCO_3$ before and after 1-hour reaction at -0.97 V_{RHE}.*

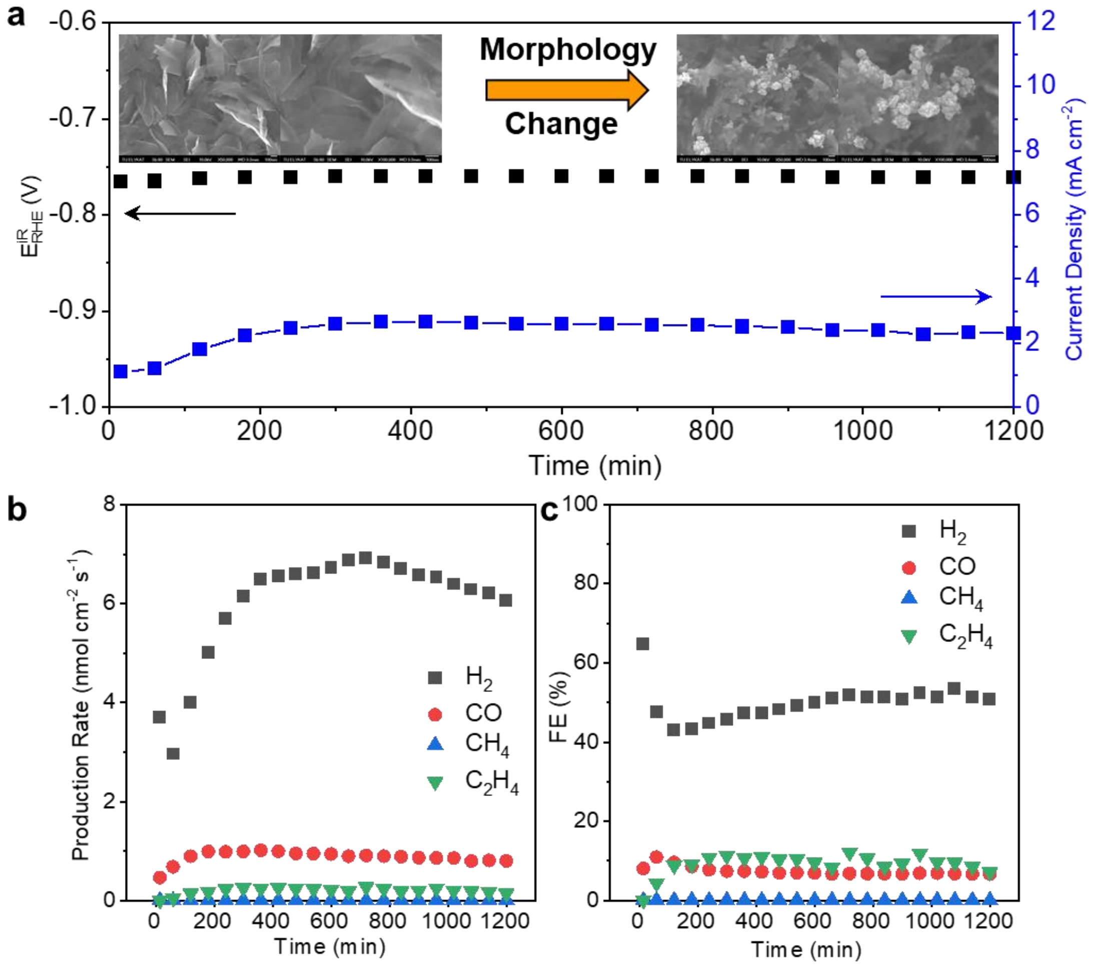

Supplementary Figure S9. *a) Chronoamperometric performance stability of the CO_2 reduction reaction on CuO NS in CO_2-saturated 0.1M $KHCO_3$ at -0.76 V_{RHE}. Insert: ex situ SEM image of as-prepared CuO catalyst on GC electrode (100 µg cm^{-2}) before and after 20-h electrolysis. b) Absolute product formation rates and c) Faradaic efficiencies of major gaseous products as a function of time during CO2RR in CO_2-saturated 0.1M $KHCO_3$ at -0.76 V_{RHE}.*

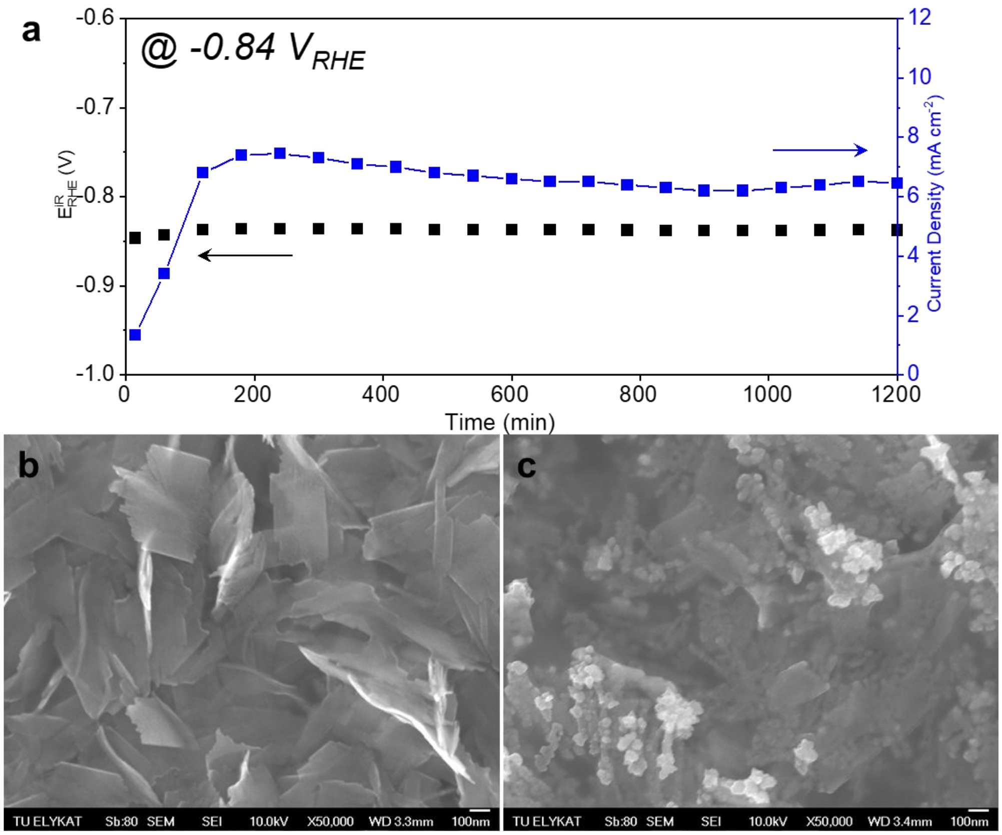

Supplementary Figure S10. *a) Chronoamperometric performance stability of the CO_2 reduction reaction on CuO NS in CO_2-saturated 0.1M $KHCO_3$ at -0.84 V_{RHE}. b) ex situ SEM images of as-prepared CuO catalyst on GC electrode (100 µg cm^{-2}) before and c) after 20-h electrolysis.*

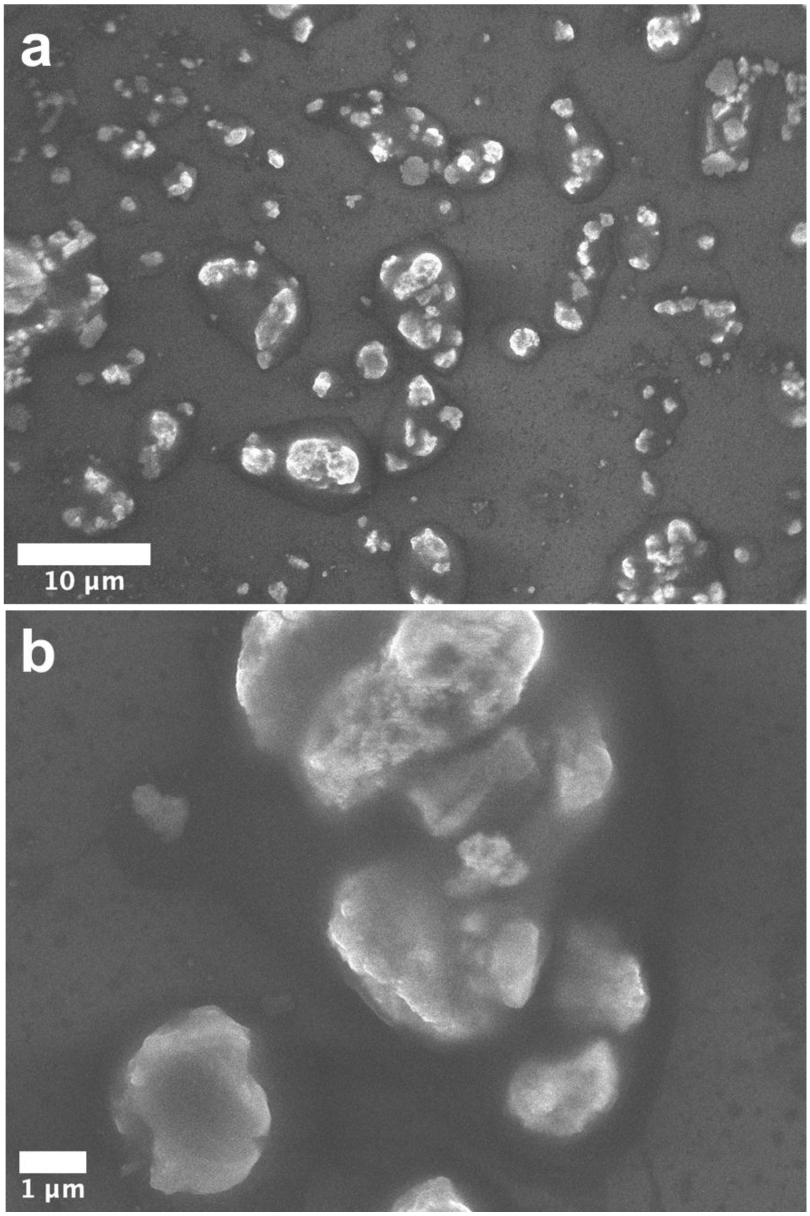

Supplementary Figure S11. *Ex situ SEM images of CuO catalyst on GC electrode (100 µg cm^{-2}) after long-term electrolysis in CO_2-saturated 0.1M $KHCO_3$ after 60 hours at -1.0 V_{RHE} in H-cell.*

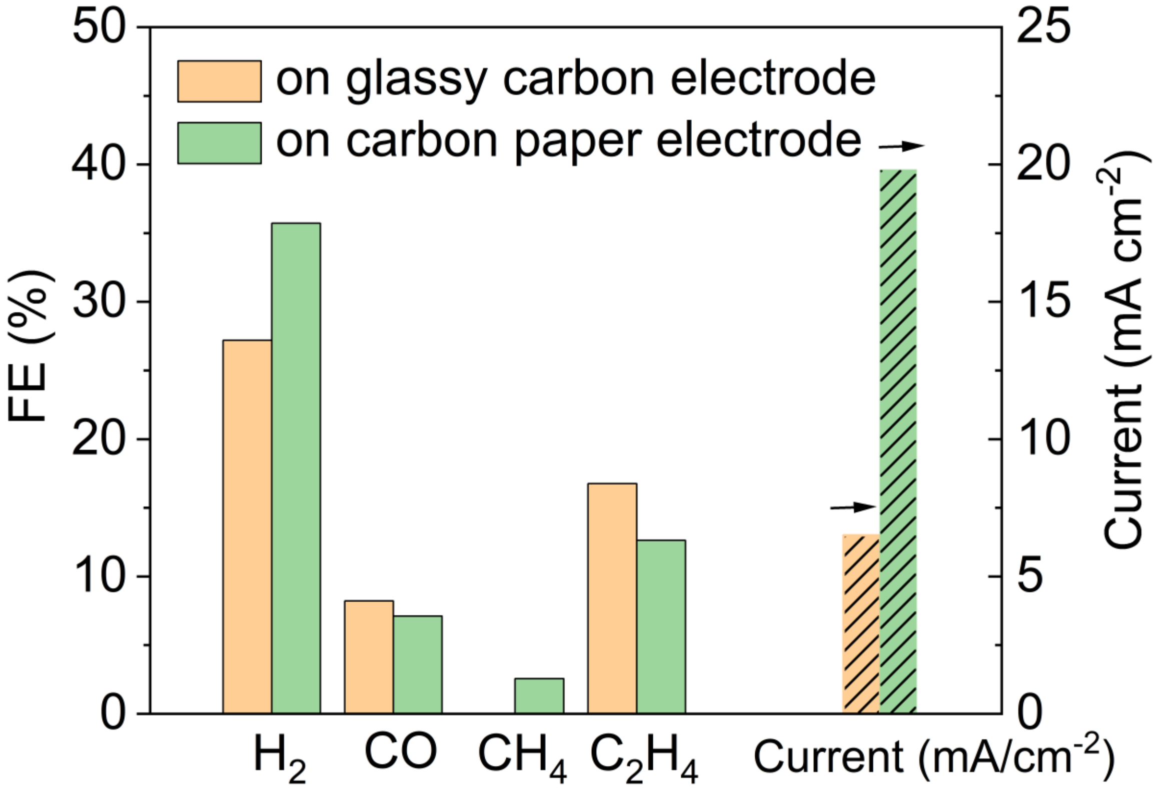

Supplementary Figure S12. *The comparison of FEs for major gaseous products and geometric current densities of CuO NS catalyst on glassy carbon electrode and carbon paper electrode after 1-hour CO2RR in CO_2-saturated 0.1M $KHCO_3$ at -0.84 V_{RHE} with same geometric catalyst loading of 100 ug/cm^2. Compared to the measurement on glassy carbon electrode, carbon paper showed a lower FE towards C_2H_4 and higher FE toward CH_4 due to the relatively lower areal particle density, which is consistent with our previous observation*[4].

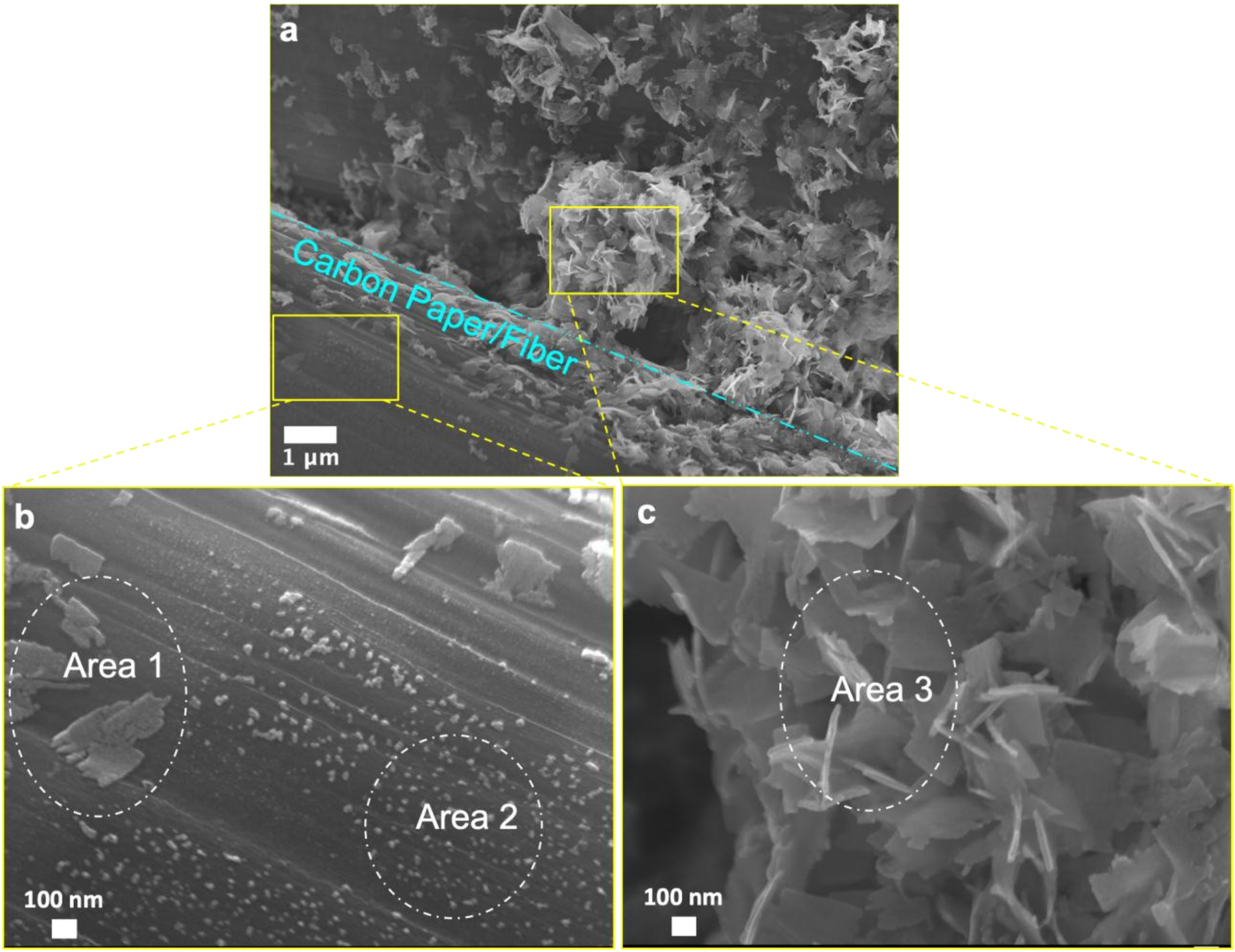

Supplementary Figure S13. *SEM images of CuO NS on carbon paper electrode after 1-h CO_2RR in H-cell at -0.84 V_{RHE} in CO_2-saturated 0.1 M $KHCO_3$ at pH 6.8 (Supplementary Figure S12). a) Large-scale image overview. b) The image with the location area directly on carbon paper electrode. Area 1: cracking CuO NS; Area 2: clusters. c) The image with the location that only connected with carbon paper electrode. Area 3: intact CuO NS.*

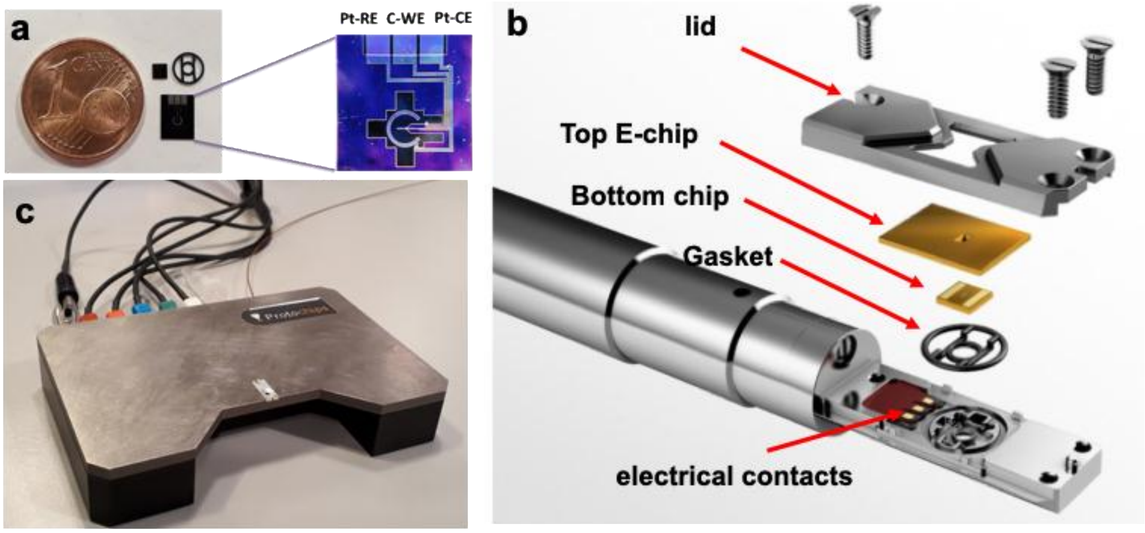

Supplementary Figure S14. *a) Photograph and dimension of gasket, the electrochemistry chips (small bottom chip and big top E-chip) with enlarged E-chip, in comparison to 1-euro cent. b) Assembling of the in situ flow-through electrochemical Echip cell with gasket, bottom chip, top E-chip and lid. c) The IL TEM cell (Protochips) with identical chip dimensions and assembly, flow mode and electrochemical connection. Catalysts tested in this cell can be imaged subsequently under dry conditions with liquid Echem holder. Images are partly adapted from "Workflow & Training Poseidon Select Version 1.2; Copyright 2017, Protochips, Inc." with permission from Protochips.*

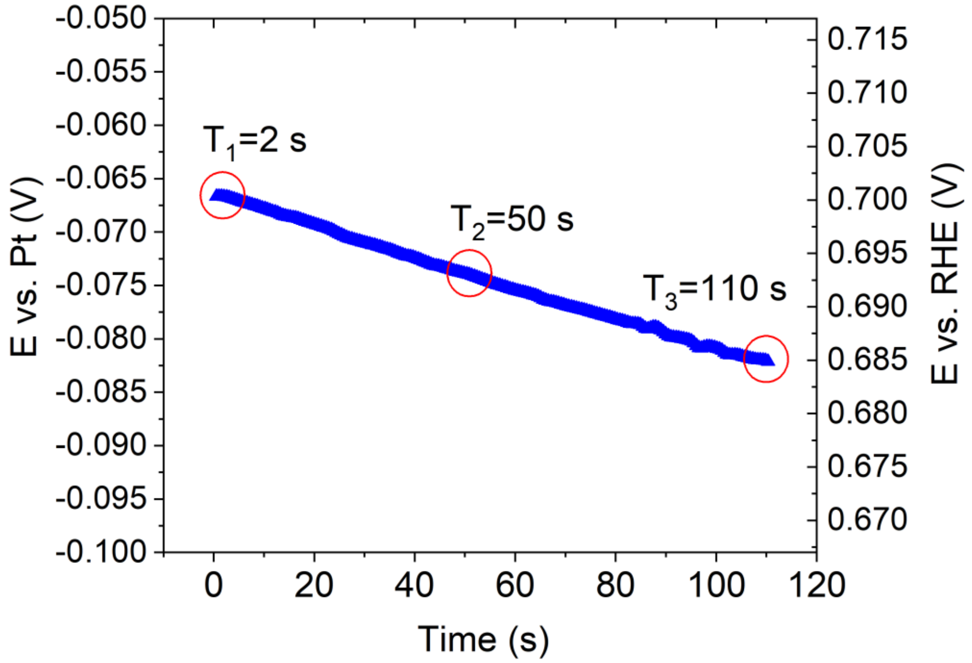

Supplementary Figure S15. *OCP profile of CuO NS in 30 uL/h H_2O flow with marked time points. The corresponding real time images are taken with in situ TEM E-chip cell and shown in Fig. 3a-b. The whole movie during this measurement is shown as Supplementary Movie 1.*

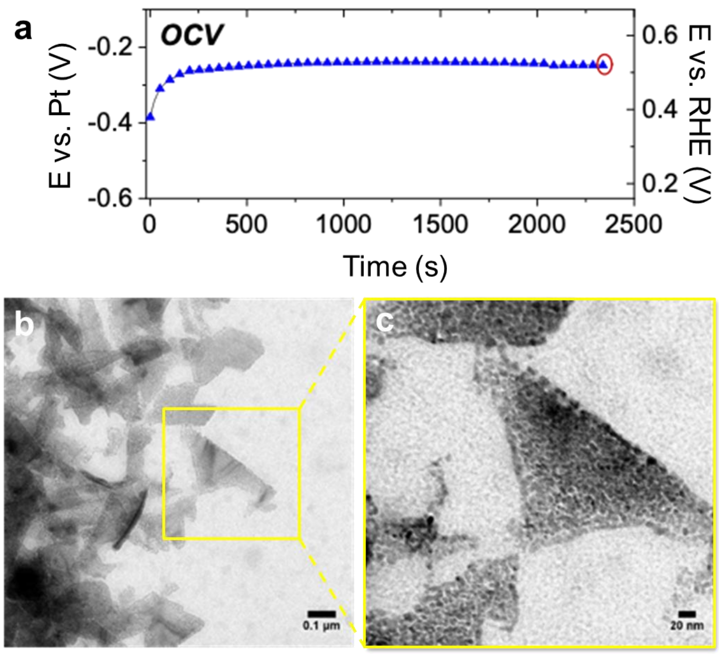

Supplementary Figure S16. *a) The OCP profile for CuO NS catalysts recorded in the IL TEM E-chip cell (displayed in Supplementary Figure S14b). b), c) TEM images of CuO NS catalysts recorded after the OCP protocol shown in Fig. S16a. The images are taken with in situ TEM E-chip cell in dry state.*

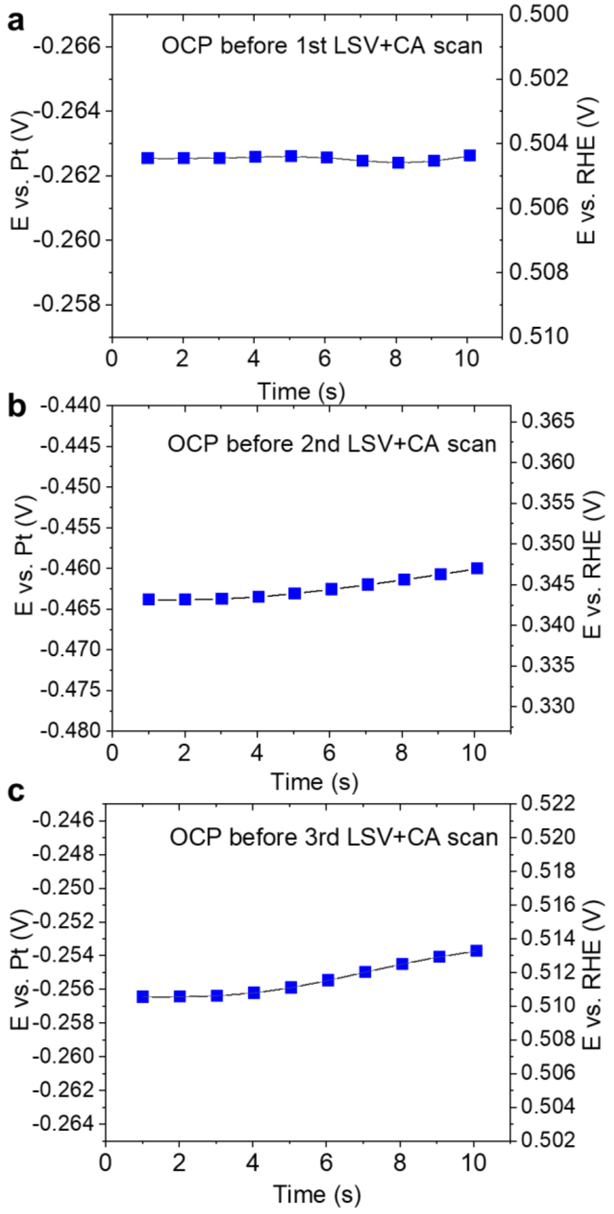

Supplementary Figure S17. *A 10-second OCP conditioning of CuO NS is recorded in a pH=6.9 buffer solution prior to LSV and CA scan (left on the pseudo Pt reference scale, right on the Reversible Hydrogen scale, RHE). a) OCP profile before 1st LSV and CA scan to E_1= -0.84 V_{RHE}. The 1st LSV+CA and corresponding snapshots are shown in Fig. 3e-h. b) OCP profile before 2nd LSV and CA scan to E_2= -1.23 V_{RHE}. The 2nd LSV+CA and corresponding snapshots are shown in Fig. 3i-j and Supplementary Figure S18. c) OCP profile before 3rd LSV and CA scan to E_2= -1.73 V_{RHE}. The 3rd LSV+CA and corresponding snapshots are shown in Fig. 3k-l and Supplementary Figure S19.*

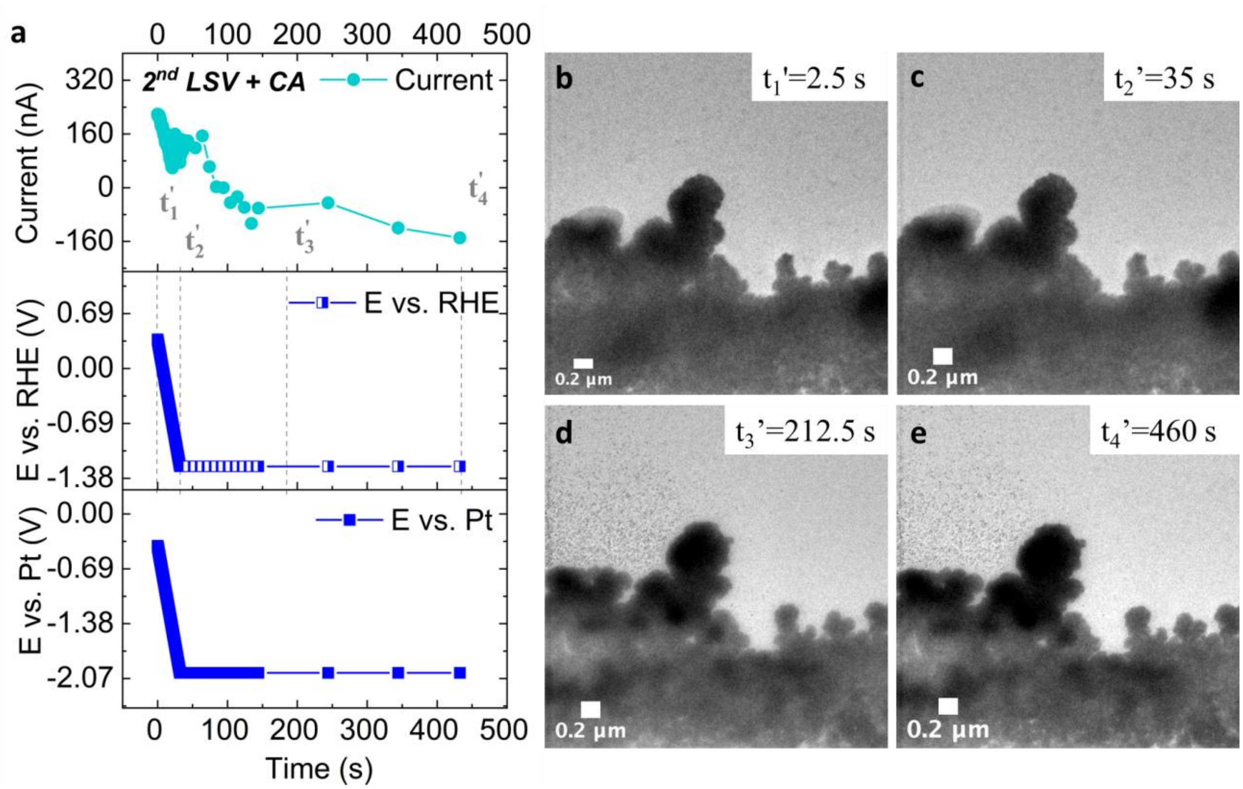

Supplementary Figure S18. *a) Current/potential profiles over time with marked time points,* t_1' *-* t_4' *(vertical lines), corresponding to the images in (b-e). Linear sweep voltammetry (LSV) is performed after 10-second OCP measurement in a pH=6.9 buffer solution flow (the OCP profile is given in Supplementary Figure S17b) with scan rate of 50 mV/s. The following Chronoamperometry (CA) are acquired at -1.23* V_{RHE} *(the second potential). The whole movie is shown as Supplementary Movie 5.*

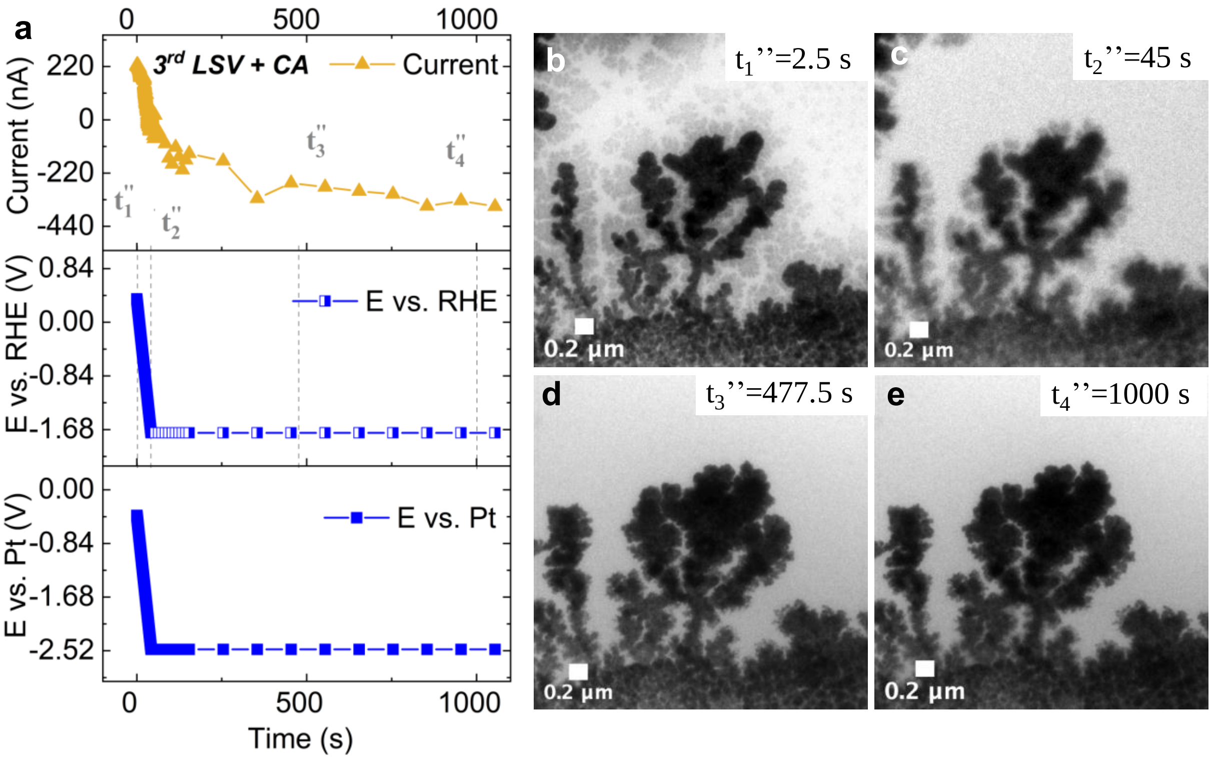

Supplementary Figure S19. *a) Current/potential profiles over time with marked time points,* t_1'' *-* t_4'' *(vertical lines), corresponding to the images in (b-e). Linear sweep voltammetry (LSV) is performed after 10-second OCP measurement in a pH=6.9 buffer solution flow (the OCP profile is given in Supplementary Figure S17c) with scan rate of 50 mV/s. The following Chronoamperometry (CA) are acquired at -1.73* V_{RHE} *(the second potential). The whole movie is shown as Supplementary Movie 6.*

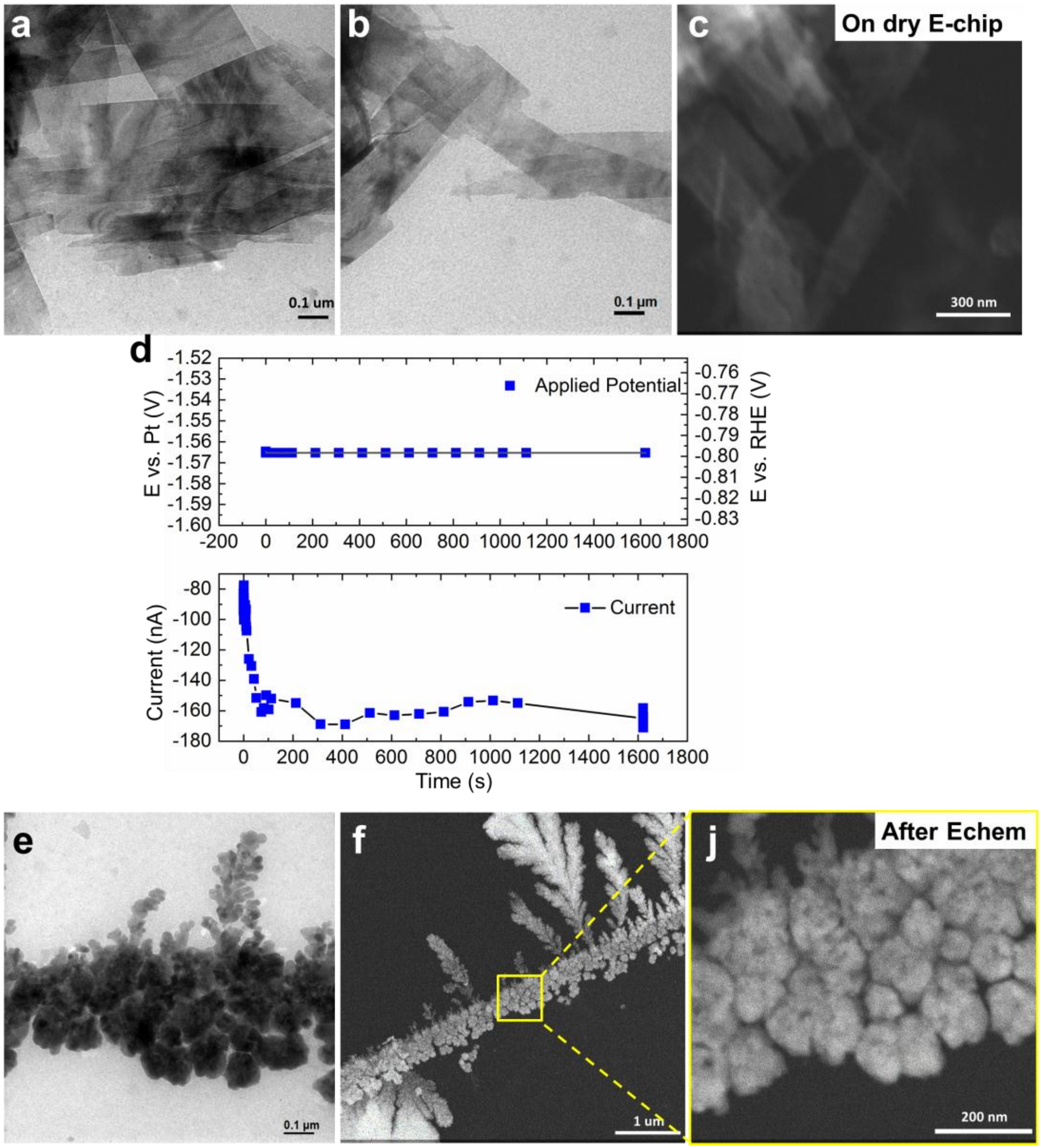

Supplementary Figure 20. *a, b) TEM and c) high-angle annular dark-field scanning transmission electron microscopy (HADDF-STEM) images of CuO NS on E-chip in dry state. d) Chronoamperometry of CuO NS at -0.80* V_{RHE} *in a pH=6.9 buffer solution for 1600s. The experiment was performed in identical location (IL) TEM E-chip cell. e) TEM and f, j) HADDF-STEM images of CuO NS after Chronoamperometry protocol. The images were taken in dry state with in situ TEM E-chip cell.*

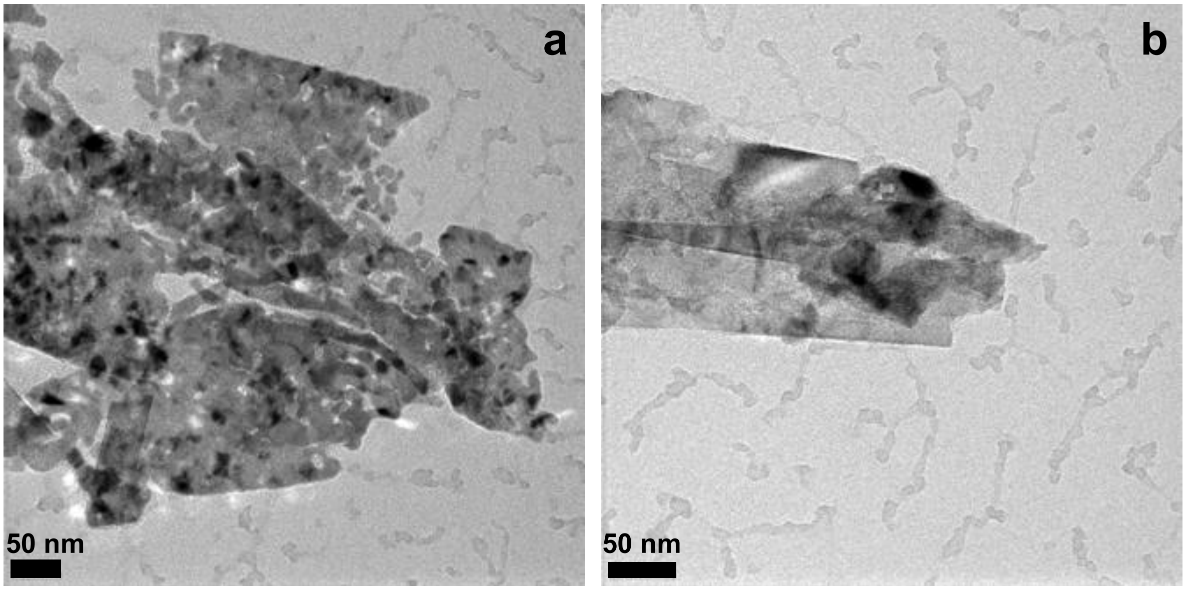

Supplementary Figure S21. *Morphology evolution of CuO NS in N_2-saturated 0.1M Na_2HPO_4/NaH_2PO_4 buffer (pH=6.9) before and after 15-min reaction at -1.0 V_{RHE}.*

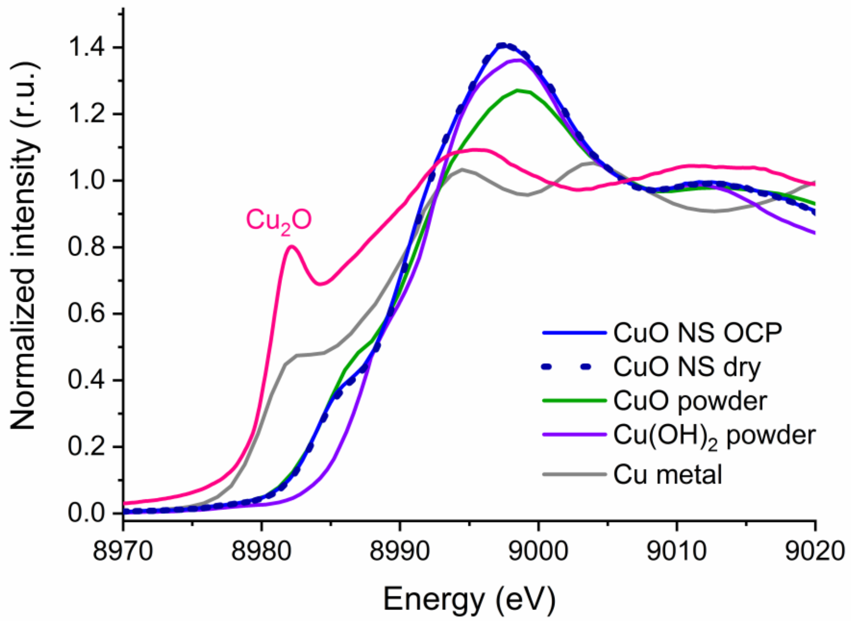

Supplementary Figure S22. *Comparison of XANES at the Cu K-edge between Cu metal, references powders CuO, Cu_2O, $Cu(OH)_2$, the CuO NS catalyst in the dry state, and at OCP in 0.1 M $KHCO_3$ at pH 6.8.*

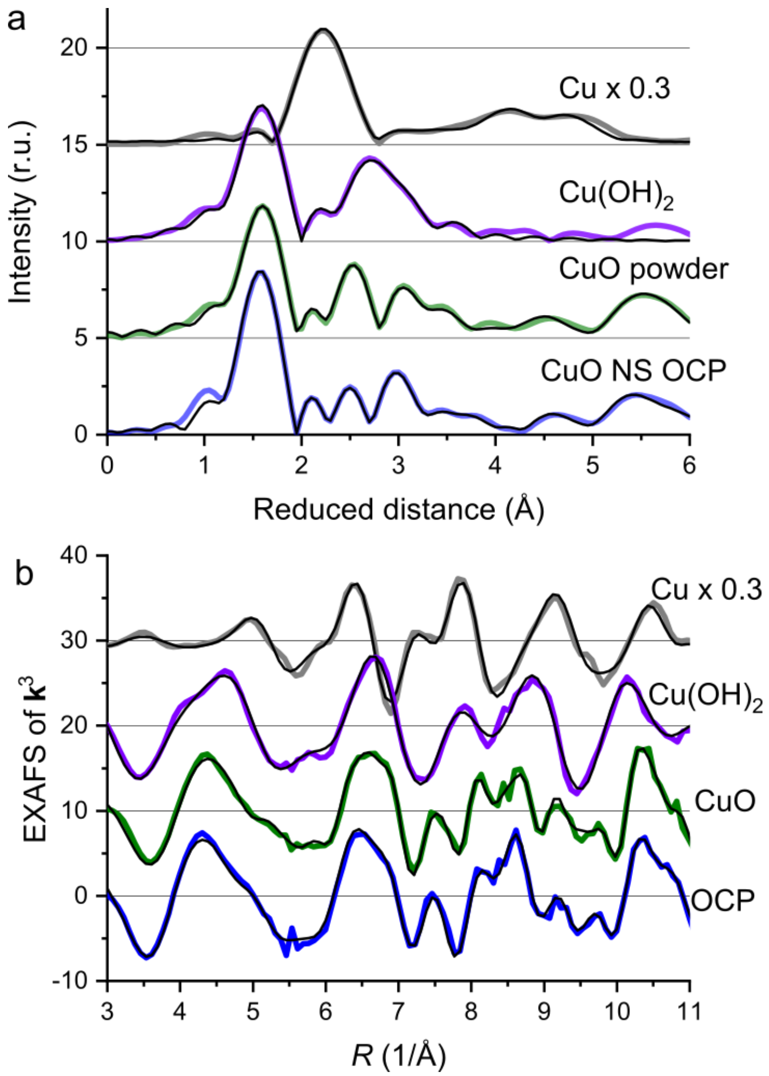

Supplementary Figure S23. *a) FT of* ***k***^{3} *weighted EXAFS and b) EXAFS at the Cu K-edge of Cu metal foil,* $Cu(OH)_2$ *powder, CuO powder, and the CuO NS catalyst at OCP in 0.1 M* $KHCO_3$ *at pH 6.8. Colored lines represent the experimental data and black lines in the simulations. The distance on the x-axis in a) is reduced by 0.35 Å relative to the real distance.*

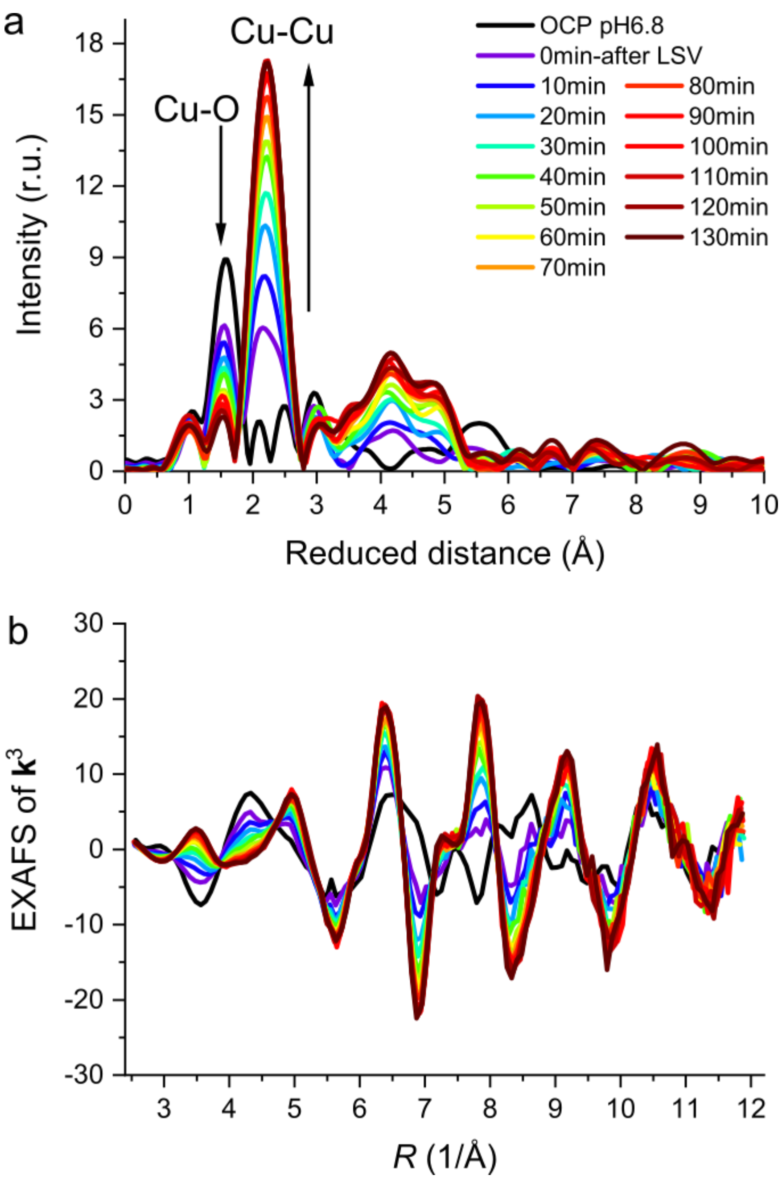

Supplementary Figure S24. *a) FT of* ***k***^{3} *weighted EXAFS at the Cu K-edge of the CuO NS film at OCP in 0.1 M* $KHCO_3$ *at pH 6.8 and during 130 min CO2RR at -0.84* V_{RHE}*. The distance on the x-axis is reduced by 0.35 Å relative to the real distance. b) EXAFS (****k***^{3} *weighted) at the Cu K-edge of the CuO NS catalyst film at OCP in 0.1 M* $KHCO_3$ *at pH 6.8 and during 130 min CO2RR at -0.84* V_{RHE}*.*

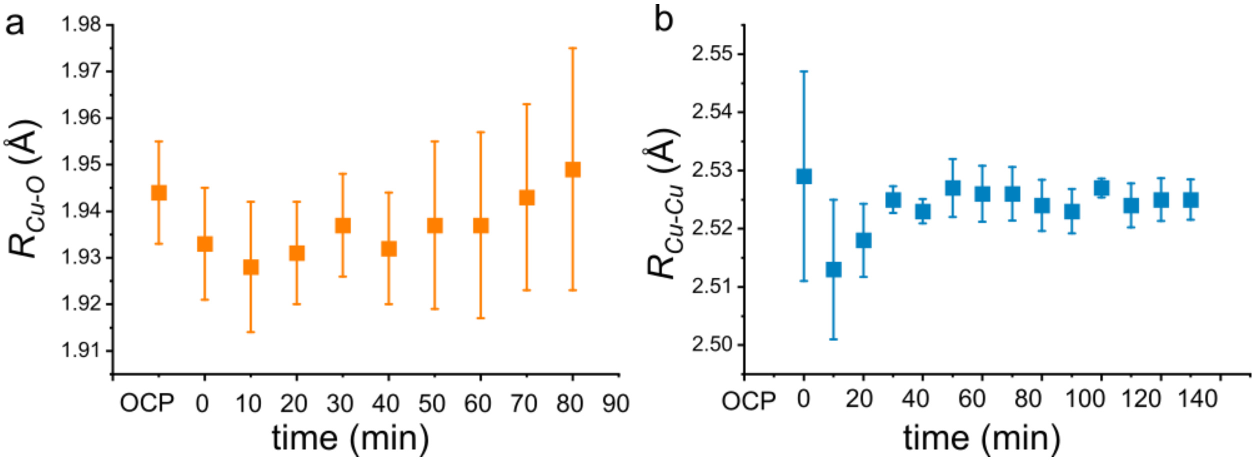

Supplementary Figure S25. *EXAFS distances of the first Cu-O coordination sphere and the first intermetallic Cu-Cu shell. The fit error was calculated as in reference*[1].

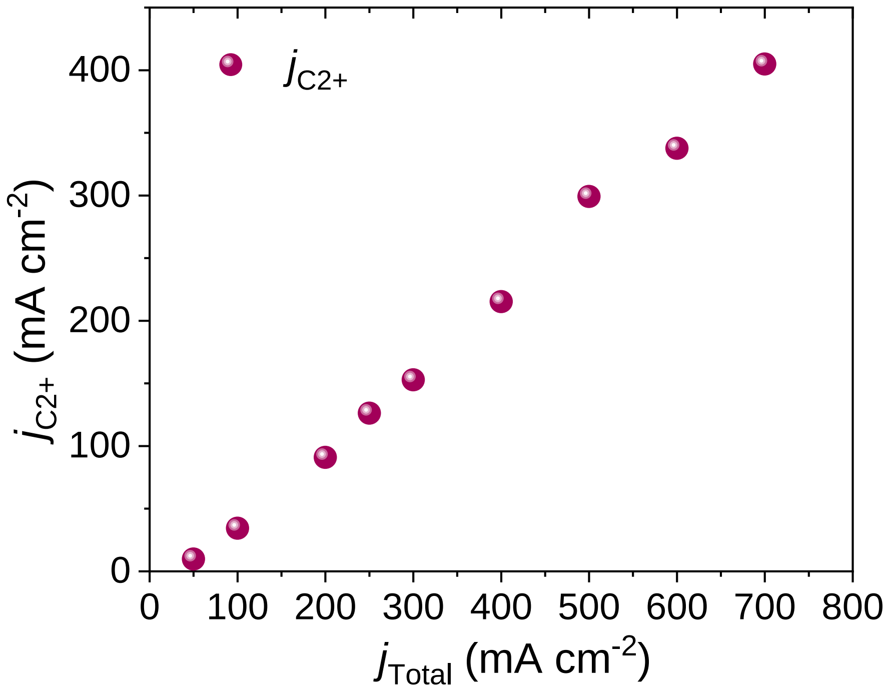

Supplementary Figure S26. *The partial current density of C_{2+} products as a function of applied geometric current density for CuO NS in 1 M $KHCO_3$.*

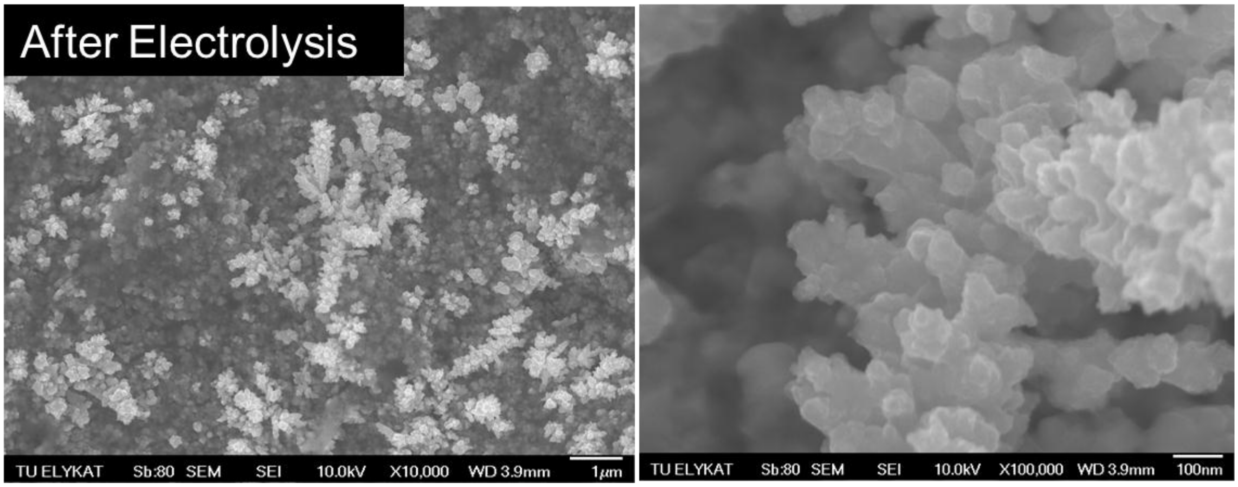

Supplementary Figure S27. *Ex situ SEM image of CuO NS GDE after* CO_2 *electrolysis for currents screening measurements in flow cell configuration.*

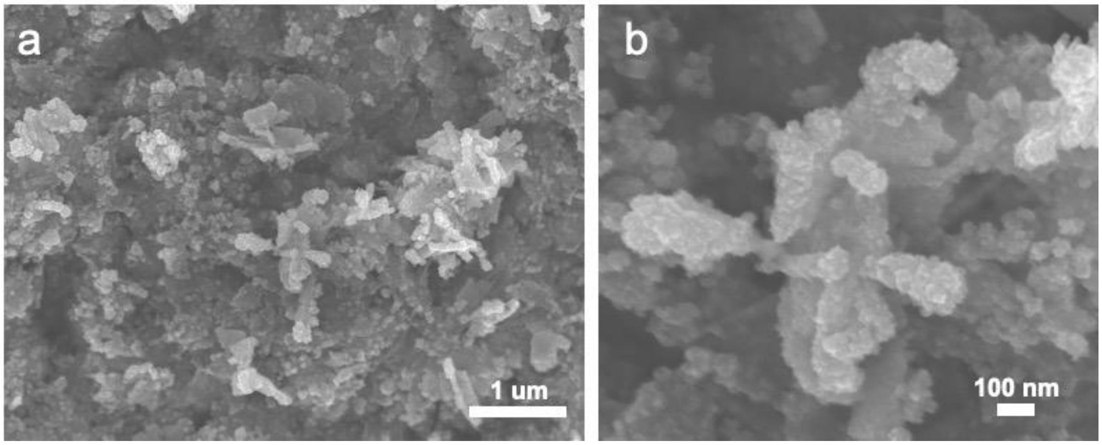

Supplementary Figure S28. *Ex situ SEM image of CuO NS GDE after CO_2 electrolysis for 24 hours at 300 mA cm^{-2} in flow cell configuration.*

Supplementary Table S1

Supplementary Table S1 *Cu K-edge simulation parameters of the FT-EXAFS oscillations of as-prepared CuO NS film. Data range k= 3-11 Å-1, Amplitude reduction factor* S_0^2*=0.8. The following simulation approach was used:* The coordination number N was fixed to the expected value (cif file), then calculated the distances R and the Debeye Waller factor for each shell were calculated, Then, the Debye Waller factor was fixed to the obtained values from before and N was calculated. This resulted in highly similar results, but slightly lower Rf factor (better fit quality). The fit error was calculated as in reference[1]. For calculation of the Fourier-filtered error (described in reference[2]), the range from 1 to 6.5 Å on the reduced distance scale was used.[1]

**CuO NS film as-prepared**					
	**Shell**	***R*** **/Å**	***CN***	**σ**	$\mathbf{R_f}$
**OCP**	Cu-O	1.94 ±0.01	4.2 ±0.13	0.0431*	11.39
	Cu-Cu	2.69 ±0.02	2.2 ±0.22	0.0415*	
	Cu-Cu	2.88 ±0.02	4.1 ±0.14	0.0629*	
	Cu-Cu	3.03 ±0.01	4.1 ±0.32	0.0536*	
	Cu-Cu	3.14 ±0.01	2.3 ±0.32	0.0318*	
	Cu-Cu	3.30 ±0.01	1.8 ±0.13	0.0997*	
**0 min**	Cu-O	1.93 ±0.01	2.82 ±0.21	0.0431*	10.19
	Cu-Cu	2.53 ±0.02	3.9 ±1	0.0853*	
	Cu-Cu	2.74 ±0.03	5.37 ±1.9	0.0629*	
	Cu-Cu	2.93 ±0.03	7.17 ±2.1	0.0536*	
	Cu-Cu	3.11 ±0.03	3.46 ±0.8	0.0318*	
	Cu-Cu	4.43 ±0.02	4.51 ±1	0.0885*	
**10 min**	Cu-O	1.93 ±0.01	2.25 ±0.22	0.0431*	11.9
	Cu-Cu	2.51 ±0.01	4.93 ±0.32	0.0853*	
	Cu-Cu	3.63 ±0.11	1.84 ±2.4	0.0906*	
	Cu-Cu	4.41 ±0.03	6.4 ±1.3	0.0885*	
	Cu-Cu	4.92 ±0.02	2.81 ±0.66	0.0344*	
	Cu-Cu	3.48 ±0.07	4.12 ±2.4	0.0997*	
**30 min**	Cu-O	1.94 ±0.01	1.56 ±0.23	0.0431*	9.62

	Shell	R /Å	CN	σ	R_f
	Cu-Cu	2.53 ±0.002	6.95 ±0.26	0.0853*	
	Cu-Cu	3.63 ±0.02	5.74 ±1.6	0.0906*	
	Cu-Cu	4.44 ±0.008	9.91 ±1.3	0.0885*	
	Cu-Cu	4.95 ±0.01	3.7 ±0.64	0.0344*	
	Cu-Cu	3.47 ±0.02	5.61 ±1.7	0.0997*	

Numbers marked with * are fixed according to the information in the cif file.

CuO NS film as-prepared

	**Shell**	***R* /Å**	***CN***	**σ**	$\mathbf{R_f}$
**50 min**	Cu-O	1.94 ±0.02	0.975 ±0.22	0.0431*	9.01
	Cu-Cu	2.53 ±0.01	8.23 ±0.26	0.0853*	
	Cu-Cu	3.61 ±0.02	7.72 ±1.6	0.0906*	
	Cu-Cu	4.44 ±0.01	11.9 ±1.3	0.0885*	
	Cu-Cu	4.95 ±0.01	4.93 ±0.65	0.0344*	
	Cu-Cu	3.45 ±0.02	6.98 ±1.8	0.0997*	
**80 min**	Cu-O	1.95 ±0.03	0.656 ±0.23	0.0431*	8.438
	Cu-Cu	2.52 ±0.004	9.23 ±0.26	0.0853*	
	Cu-Cu	3.64 ±0.02	8.44 ±1.6	0.0906*	
	Cu-Cu	4.44 ±0.01	14.4 ±1.3	0.0885*	
	Cu-Cu	4.96 ±0.01	5.63 ±0.64	0.0344*	
	Cu-Cu	3.46 ±0.02	6.88 ±1.7	0.0997*	
**90 min**	Cu-Cu	2.523 ±0.004	9.74 ±0.25	0.0853*	12.63
	Cu-Cu	3.58 ±0.02	2.8 ±0.7	0.0906*	
	Cu-Cu	4.44 ±0.008	14.9 ±1.2	0.0885*	
	Cu-Cu	4.96 ±0.009	5.95 ±0.62	0.0344*	
**120 min**	Cu-Cu	2.53 ±0.004	10.1 ±0.25	0.0853*	12.1
	Cu-Cu	3.61 ±0.01	3.91 ±0.27	0.0906*	
	Cu-Cu	4.44 ±0.01	14.8 ±1.2	0.0885*	
	Cu-Cu	4.95 ±0.01	6.54 ±0.62	0.0344*	

Only when the Cu metal content is ≤90% it is reasonable to include CuO distances (marked red)

Reference materials					
	**Shell**	***R*** **/Å**	***CN***	**σ**	R_f
**CuO powder**	Cu-O	1.95 ± 0.01	4*	0.0614	9.07
	Cu-O	2.74 ± 0.01	2*	0.0997	
	Cu-Cu	2.88 ± 0.02	4*	0.081	
	Cu-Cu	3.02 ± 0.01	4*	0.0717	
	Cu-Cu	3.16 ± 0.02	2*	0.0317	
	Cu-Cu	3.38 ± 0.03	2*	0.99	
	Cu-Cu	3.88 ± 0.03	2*	0.0998	
	Cu-Cu	4.79 ± 0.01	5*	0.0747	
	Cu-Cu	4.97 ± 0.01	4*	0.0333	
**$Cu(OH)_2$ powder**	Cu-O	1.97 ± 0.01	2.2 ± 0.18	0.0317*	10.2
	Cu-O	1.89 ± 0.02	2.2 ± 0.17	0.0596*	
	Cu-O	2.89 ± 0.02	2.11 ± 0.27	0.0987*	
	Cu-Cu	2.93 ± 0.03	2.08 ± 0.15	0.0316*	
	Cu-Cu	3.33 ± 0.03	3.68 ± 0.16	0.0987*	
**Cu metal**	Cu-Cu	2.53 ± 0.001	12 ± 0.24	0.0853*	10.87
	Cu-Cu	3.60 ± 0.01	5.57 ± 0.21	0.0906*	
	Cu-Cu	4.44 ± 0.004	21 ± 1.2	0.0885*	
	Cu-Cu	4.96 ± 0.004	8.65 ± 0.59	0.0344*	

Supplementary Table S2. *Comparison of DEMS-derived onset potentials for main products.*

**Cycle Number**	**E_{onset}-CO (RHE)**	**E_{onset}-CH_4 (RHE)**	**E_{onset}-C_2H_4 (RHE)**	**E_{onset}-EtOH (RHE)**
1	-0.38 ±0.08	-0.86 ±0.01	-0.57 ±0.07	-0.79 ±0.02
5	-0.43 ±0.10	-0.75 ±0.02	-0.61 ±0.06	-0.75 ±0.06
10	-0.48 ±0.05	-0.78 ±0.04	-0.63 ±0.09	-0.75 ±0.09
20	-0.44 ±0.08	-0.86 ±0.03	-0.59 ±0.05	-0.69 ±0.08
30	-0.50 ±0.11	-0.86 ±0.02	-0.69 ±0.06	-0.82 ±0.03
50	-0.43 ±0.07	-0.88 ±0.02	-0.71 ±0.05	-0.84 ±0.04
75	-0.56 ±0.08	-0.89 ±0.02	-0.74 ±0.06	-0.89 ±0.03
100	-0.48 ±0.03	-0.90 ±0.01	-0.77 ±0.02	-0.87 ±0.03

All raw data are available upon request.

References:

1. Risch, M. *et al.* Nickel-oxido structure of a water-oxidizing catalyst film. *Chem.Commun.*2011, **47**(43)**:** 11912-11914.

2. Risch, M. *et al.* Water oxidation by amorphous cobalt-based oxides: in situ tracking of redox transitions and mode of catalysis. *Energy & Environ. Sci.* 2015, **8**(2)**:** 661-674.

3. Cudennec, Y., Lecerf, A. The transformation of $Cu(OH)_2$ into CuO, revisited. *Solid State Sci.* 2003, **5**(11)**:** 1471-1474.

4. Wang, X. *et al.* Catalyst Particle Density Controls Hydrocarbon Product Selectivity in CO_2 Electroreduction on CuO_x. *ChemSusChem* 2017, **10**(22)**:** 4642-4649.
