# Supplementary material for: Morphology and mechanism of highly selective Cu(II) oxide nanosheet catalysts for carbon dioxide electroreduction
Source: Nat Commun. 2021 Feb 4;12:794. doi: 10.1038/s41467-021-20961-7 (PMC7862240; doi:10.1038/s41467-021-20961-7)
Supplement: Supplementary file 2 — Description of Additional Supplementary Files [file 41467_2021_20961_MOESM2_ESM.pdf]

Description of Additional Supplementary Files

Supplementary Movie 1 Evolution of CuO NS under OCP. The video was taken under OCP (see Supplementary Figure 15) in 30 uL/h H_2O. Selected time points with TEM snapshots are shown in Fig. 3a-c.

Supplementary Movie 2 Evolution of CuO NS under liquid electrolyte feed changing. The electrolyte feed in in situ TEM E-chip cell was changed was changed to a pH=6.9 buffer solution during the 200s.

Supplementary Movie 3 Evolution of CuO NS under liquid electrolyte feed changing. The electrolyte feed in in situ TEM E-chip cell was changed was continously changed to a pH=6.9 buffer solution during the 198s.

Supplementary Movie 4 Evolution of CuO NS under 1st LSV+CA. Linear sweep voltammetry (LSV) is performed in a pH=6.9 buffer solution flow with scan rate of 50 mV/s. The following Chronoamperometry (CA) are holed at -0.84 V_{RHE} (the first potential).

Supplementary Movie 5 Evolution of CuO NS under 2nd LSV+CA. Linear sweep voltammetry (LSV) is performed in a pH=6.9 buffer solution flow with scan rate of 50 mV/s. The following Chronoamperometry (CA) are holed at -1.23 V_{RHE} (the second potential).

Supplementary Movie 6 Evolution of CuO NS under 3rd LSV+CA. Linear sweep voltammetry (LSV) is performed in a pH=6.9 buffer solution flow with scan rate of 50 mV/s. The following Chronoamperometry (CA) are holed at -1.73 V_{RHE} (the third potential).
